# Single-Atom Iridium on Hematite Photoanodes for Solar Water Splitting: Catalyst or Spectator?

**DOI:** 10.1021/jacs.2c09974

**Published:** 2023-01-11

**Authors:** Qian Guo, Qi Zhao, Rachel Crespo-Otero, Devis Di Tommaso, Junwang Tang, Stoichko D. Dimitrov, Maria-Magdalena Titirici, Xuanhua Li, Ana Belén Jorge Sobrido

**Affiliations:** † School of Engineering and Materials Science, Queen Mary University of London, E1 4NS London, U.K.; ‡ School of Physical and Chemical Sciences, Queen Mary University of London, E1 4NS London, U.K.; § Department of Chemical Engineering, University College London, Torrington Place, WC1E 7JE London, U.K.; ∥ Department of Chemical Engineering, Imperial College London, SW7 2AZ London, U.K.; ⊥ State Key Laboratory of Solidification Processing, Center for Nano Energy Materials, School of Materials Science and Engineering, Northwestern Polytechnical University, 710072 Xi’an, China

## Abstract

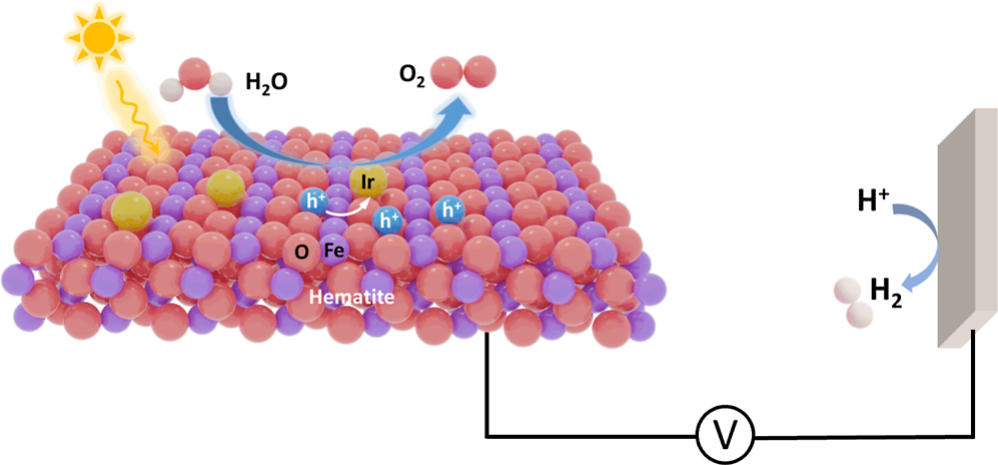

Single-atom catalysts (SACs) on hematite photoanodes are efficient cocatalysts to boost photoelectrochemical performance. They feature high atom utilization, remarkable activity, and distinct active sites. However, the specific role of SACs on hematite photoanodes is not fully understood yet: Do SACs behave as a catalytic site or as a spectator? By combining spectroscopic experiments and computer simulations, we demonstrate that single-atom iridium (sIr) catalysts on hematite (α-Fe_2_O_3_/sIr) photoanodes act as a true catalyst by trapping holes from hematite and providing active sites for the water oxidation reaction. In situ transient absorption spectroscopy showed a reduced number of holes and shortened hole lifetime in the presence of sIr. This was particularly evident on the second timescale, indicative of fast hole transfer and depletion toward water oxidation. Intensity-modulated photocurrent spectroscopy evidenced a faster hole transfer at the α-Fe_2_O_3_/sIr/electrolyte interface compared to that at bare α-Fe_2_O_3_. Density functional theory calculations revealed the mechanism for water oxidation using sIr as a catalytic center to be the preferred pathway as it displayed a lower onset potential than the Fe sites. X-ray photoelectron spectroscopy demonstrated that sIr introduced a mid-gap of 4d state, key to the fast hole transfer and hole depletion. These combined results provide new insights into the processes controlling solar water oxidation and the role of SACs in enhancing the catalytic performance of semiconductors in photo-assisted reactions.

## Introduction

Photoelectrochemical (PEC) water splitting represents a sustainable and cost-effective route to convert solar energy directly into chemical energy in the form of molecular hydrogen.^1−4^ Hematite (α-Fe_2_O_3_) has been targeted as one of the most promising metal-oxide photoanodes in PEC configuration due to its natural abundance, effective use of visible light, and excellent photo and chemical stability.^5^ However, hematite photoanodes still underperform in terms of solar-to-hydrogen efficiency, far below its corresponding theoretical value.^6^ The sluggish four-electron-transfer water oxidation reaction is one of the main reasons for the lower efficiency of hematite photoanodes.^4,7^ To facilitate the water oxidation process, surface modification of hematite via decoration with a suitable cocatalyst has been proposed as a promising strategy to lower the reaction barrier.^8−10^ Although several cocatalysts such as IrO_*x*_,^6^ Co–Pi (Pi = phosphate),^11^ and NiFeO_*x*_^12^ can substantially improve the PEC performance of hematite photoanodes, their role in the mechanism of water oxidation reaction is still under debate. Transient absorption spectroscopy (TAS) of hematite decorated with the Co–Pi cocatalyst showed that the hematite/Co–Pi heterojunction reduced the charge recombination by increasing band bending instead of improving the water oxidation kinetics through hole transfer to Co–Pi.^13^ In contrast, steady-state and transient PEC measurements and impedance spectroscopic investigation of Co–Pi-coated hematite photoanodes clearly demonstrated efficient hole transfer from hematite to Co–Pi and that water oxidation occurred predominately from the Co–Pi film, not the hematite surface, which accelerated the water oxidation efficiency and hence improved the water-splitting performance.^14^ A kinetic study of NiFeO_*x*_-modified hematite photoanodes by intensity-modulated photocurrent spectroscopy (IMPS) suggested a passivation function of NiFeO_*x*_ on hematite,^15^ while a bifunctional role of hole storage and catalytic activity of NiFeO_*x*_ on hematite was identified by double-working electrode measurements.^16^ As such, finding a cocatalyst that directly boosts water oxidation on its active sites and knowing its working mechanism is critical for the development of efficient photoanodes.

Using single-atom catalysts (SACs) with atomically distributed metal sites on supports is an innovative approach to maximize the photo-electrocatalytic activity of a semiconductor. Even though only a few attempts have reported on the integration of SACs with hematite photoanodes, their excellent performance validates the feasibility and potential of the approach. For example, single nickel on α-Fe_2_O_3_ photoanodes, supported on ultrathin carbon nanosheets, led to a photocurrent density of 1.85 mA cm^–2^ at 1.23 V versus the reversible hydrogen electrode (RHE), a 2.2-fold enhancement compared to pure α-Fe_2_O_3_.^17^ Similarly, single-atom Ir directly bonded on α-Fe_2_O_3_ delivered a high photocurrent density of 1.01 mA cm^–2^ at 1.23 V versus RHE with a particularly low value for the onset potential of 0.63 V versus RHE at a pH of 6.0.^18^

However, despite remarkable progress achieved, the lack of fundamental and systematic mechanistic investigations of such systems limits our understanding of the specific function of SAC on α-Fe_2_O_3_ photoanodes, that is, whether any enhanced activity results from a specific SAC catalytic effect or by retardation of recombination kinetics. To reveal the role of SACs in enhancing the PEC activity of hematite anodes, we have conducted experiments (in situ TAS, IMPS, and ultraviolet photoelectron spectroscopy) and simulations (DFT) of water oxidation on single-atom iridium (sIr) directly bonded to α-Fe_2_O_3_ photoanodes (α-Fe_2_O_3_/sIr).

## Results and Discussion

### Synthesis and Characterization of α-Fe_2_O_3_/sIr

The molecular Ir catalyst (mIr) [2-(pyridine-2yl)-2-propanato iridium(IV) dimer], which has low overpotential, high turnover frequency, and minimal degradation for water oxidation,^19^ can directly and robustly bind to oxide surfaces. This Ir dimer was employed here for the synthesis of α-Fe_2_O_3_/sIr via a previously reported heterogenization method followed by a room-temperature photochemical treatment for ligand removal.^18^ As illustrated in Scheme S1, mIr was loaded onto hematite (α-Fe_2_O_3_/mIr) by submerging a hematite photoelectrode into an mIr aqueous solution overnight. The mIr cocatalyst then underwent a photochemical treatment (Figure S1). This process led to the decomposition of the molecular ligands and generated single Ir atoms on hematite photoanodes (α-Fe_2_O_3_/sIr).

**Figure 1:**
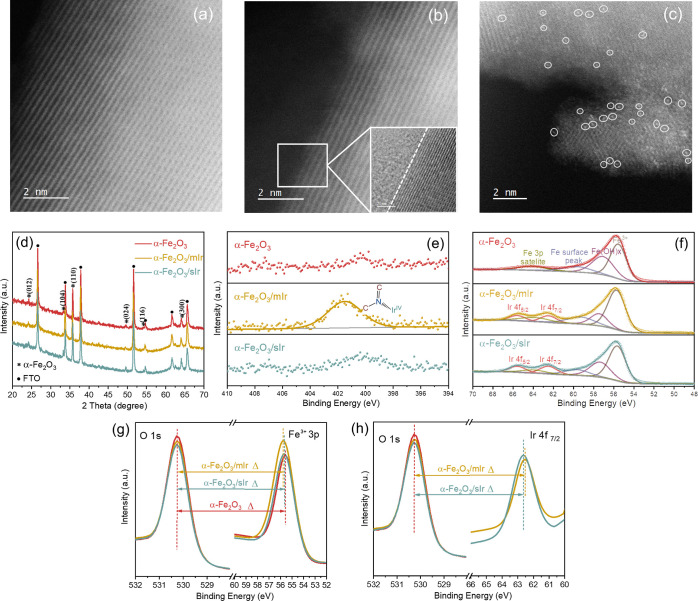
Representative HAADF-STEM micrographs of (a) α-Fe_2_O_3_, (b) α-Fe_2_O_3_/mIr, and (c) α-Fe_2_O_3_/sIr; (d) XRD profiles of α-Fe_2_O_3_, α-Fe_2_O_3_/mIr, and α-Fe_2_O_3_/sIr; high-resolution XPS spectra of (e) N 1s and (f) Fe 3p and Ir 4f for α-Fe_2_O_3_, α-Fe_2_O_3_/mIr, and α-Fe_2_O_3_/sIr and of (g) O 1s and Fe^3+^ 3p and (h) O 1s and Ir 4f_7/2_ for α-Fe_2_O_3_, α-Fe_2_O_3_/mIr, and α-Fe_2_O_3_/sIr.

The surface morphology and sIr dispersion of the as-prepared α-Fe_2_O_3_/sIr were assessed by scanning transmission electron microscopy (STEM). As shown in Figure 1a, α-Fe_2_O_3_ exhibited highly defined crystalline planes. In comparison, the α-Fe_2_O_3_/mIr intermediate material (Figure 1b) exhibited an amorphous layer on the hematite surface, attributed to the organic ligands present in the Ir molecular catalyst. Because the amorphous layer covers the Ir atoms, microscopy cannot detect these atoms.^18^ In the case of α-Fe_2_O_3_/sIr, isolated bright dots (marked in white circles) representing single Ir atoms are observed on the hematite surface (Figure 1c). More data of the recorded Ir SAC on hematite by high-angle annular dark-field STEM (HAADF-STEM) are provided in Figure S2a–i. In these images, approximately 200 Ir units were observed. Ir SAC takes up 79% of all observed Ir units (Figure S2j), evidencing that the Ir atoms are mostly dispersed individually onto the hematite surface. The sharp peak in the HAADF intensity profile (Figure S2k) taken along the atoms of α-Fe_2_O_3_/sIr surface, assigned to individual Ir atoms, further confirmed the existence of single Ir atoms. A longer 35 min photochemical treatment resulted in the aggregation of the Ir atoms, as shown in Figure S3. The optimal time to convert mIr into sIr via photochemical treatment without undergoing aggregation of the Ir atoms was found to be 25 min.

Figure 1d shows the X-ray diffraction (XRD) pattern of α-Fe_2_O_3_, α-Fe_2_O_3_/mIr, and α-Fe_2_O_3_/sIr on fluorine-doped tin oxide (FTO)-coated glass. The diffraction peaks at 24.2, 33.2, 35.7, 49.6, 54.2, and 64.1° are assigned to (012), (104), (110), (024), (116), and (300) planes of hematite (α-Fe_2_O_3_: JCPDS no. 33-0664), respectively. The rest of the peaks are attributed to the FTO substrate (SnO_2_: JCPDS no. 77-0452). (110) exhibits the most intense diffraction peak, indicating a preferred orientation along the [110] direction, in agreement with a previous work.^12^ In combination with the crystal lattice analysis of the synthesized α-Fe_2_O_3_ from HAADF-STEM images (Figure S4), the (110) facet was determined to be one of the dominant facets of the synthesized hematite. The (110) facet has been previously reported as the primary and most active for water oxidation due to the highest conduction along this direction.^20,21^ No additional diffraction peaks relating to Ir/IrO_*x*_ particles were found in the samples of α-Fe_2_O_3_/mIr and α-Fe_2_O_3_/sIr. This result further confirms the highly dispersed nature of Ir on the hematite surface.

X-ray photoelectron spectroscopy (XPS) was conducted to examine the element composition and element chemical state on the surface of α-Fe_2_O_3_, α-Fe_2_O_3_/mIr, and α-Fe_2_O_3_/sIr (Figure S5). A clear doublet peak of Ir 4f located at 64.9 eV and singlet peaks of Ir 4d at 313.4 and 298.9 eV, separately, were observed for α-Fe_2_O_3_/mIr and α-Fe_2_O_3_/sIr samples. The surface element percentage of each sample in Table S1 shows an almost identical Ir/Fe ratio, that is, 2.22 and 2.26% for α-Fe_2_O_3_/mIr and α-Fe_2_O_3_/sIr, respectively, confirming that the Ir content remains constant after the photochemical conversion of mIr into sIr. High-resolution C 1s XPS spectra of each sample are given in Figure S6. Figure 1e presents the core-level XPS spectra of N 1s for each studied sample. No peaks in the N 1s spectra were observed for α-Fe_2_O_3_ and α-Fe_2_O_3_/sIr, whereas α-Fe_2_O_3_/mIr exhibited an intense peak at 400.6 eV assigned to the C–N–Ir structure in mIr. A typical doublet peak from Ir 4f was found for the α-Fe_2_O_3_/mIr and α-Fe_2_O_3_/sIr samples in addition to the peak of Fe 3p and its satellite peak (64.4 eV) (Figure 1f), further confirming the presence of Ir in α-Fe_2_O_3_/mIr and α-Fe_2_O_3_/sIr samples. Furthermore, the split peaks of Ir 4f, Ir 4f_5/2_, and Ir 4f_7/2_, located at around 65.7 and 62.6 eV, respectively, revealed that Ir existed as Ir^4+^ in both α-Fe_2_O_3_/mIr and α-Fe_2_O_3_/sIr.^19^

In order to identify the change in the chemical state of surface Fe before and after loading of the Ir species as well as that of Ir before and after the photochemical process, the Fe^3+^ 3p and Ir 4f_7/2_ components were plotted with respect to the O 1s oxide peak (O^2–^).^22^ As shown in Figure 1g, the peaks of O^2–^ of each sample are located at the same position. The separation of the O^2–^ and Fe^3+^ 3p peaks (indicated by Δ) for α-Fe_2_O_3_/mIr (474.50 eV) and α-Fe_2_O_3_/sIr (474.60 eV) is 0.2 and 0.1 eV, respectively, smaller than that observed for α-Fe_2_O_3_ (474.70 eV), representing an up-shift toward higher binding energy of the Fe^3+^ 3p peaks in α-Fe_2_O_3_/mIr and α-Fe_2_O_3_/sIr. This confirmed the strong interaction between sIr and α-Fe_2_O_3_, in which Fe acted as an electron-donating site and Ir as an electron-accepting site. The 0.1 eV up-shift of the Ir 4f_7/2_ peak (Figure 1h) for α-Fe_2_O_3_/sIr as compared to that forα-Fe_2_O_3_/mIr further confirms that the coordination environment of sIr is different from that of mIr because of the decomposition of ligands by photochemical treatment.

### PEC Properties of α-Fe_2_O_3_/sIr

**Figure 2:**
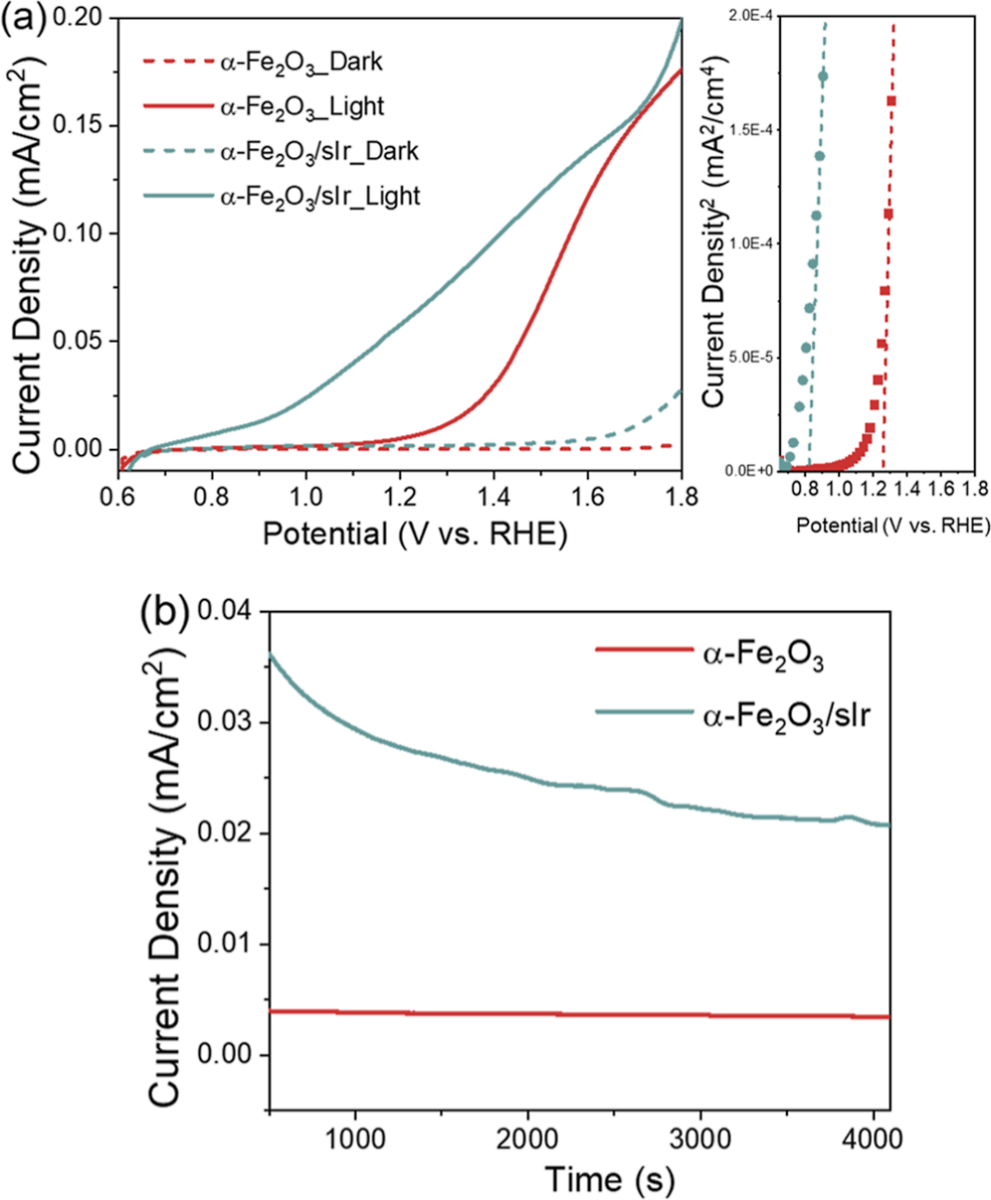
(a) LSV of α-Fe_2_O_3_ and α-Fe_2_O_3_/sIr under dark and illumination at visible light of 480 nm in 0.1 M KNO_3_ solution with a pH of 7.0 (left) and butler plots (right), in which the onset potential is defined as the value at which the extrapolation of the linear function of the current density^2^ vs potential intercepts with the current density^2^ = 0;^23^ (b) chronoamperometry profile of α-Fe_2_O_3_ and α-Fe_2_O_3_/sIr at 1.2 V vs RHE.

The PEC properties of α-Fe_2_O_3_/sIr were examined by linear sweep voltammetry (LSV) measurements under dark and light illumination at a visible light of 480 nm in 0.1 M KNO_3_ solution with a pH of 7.0. As shown in Figure 2a, the α-Fe_2_O_3_/sIr delivered a low onset potential (determined by butler plots, Figure 2a right) of ∼0.82 V versus RHE,^23^ which cathodically shifted 0.44 V from 1.26 V versus RHE for α-Fe_2_O_3_. In addition, the photocurrent of α-Fe_2_O_3_/sIr was significantly improved with respect to α-Fe_2_O_3_, particularly at lower potentials. For example, the photocurrent of α-Fe_2_O_3_/sIr at 1.2 V versus RHE is 57.5 μA cm^–2^, which is 10 times higher than that of α-Fe_2_O_3_ under the same applied bias (4.4 μA cm^–2^).

The improved PEC performance of α-Fe_2_O_3_/sIr demonstrated the great influence of sIr to enhance the photocatalytic activity of hematite for water oxidation. Figure 2b shows the chronoamperometry profile of α-Fe_2_O_3_ and α-Fe_2_O_3_/sIr. For the α-Fe_2_O_3_/sIr, the gradual decay with time of the photocurrent density is likely caused by sIr detachment from the hematite surface.^23^

### Hole Kinetics of α-Fe_2_O_3_/sIr

**Figure 3:**
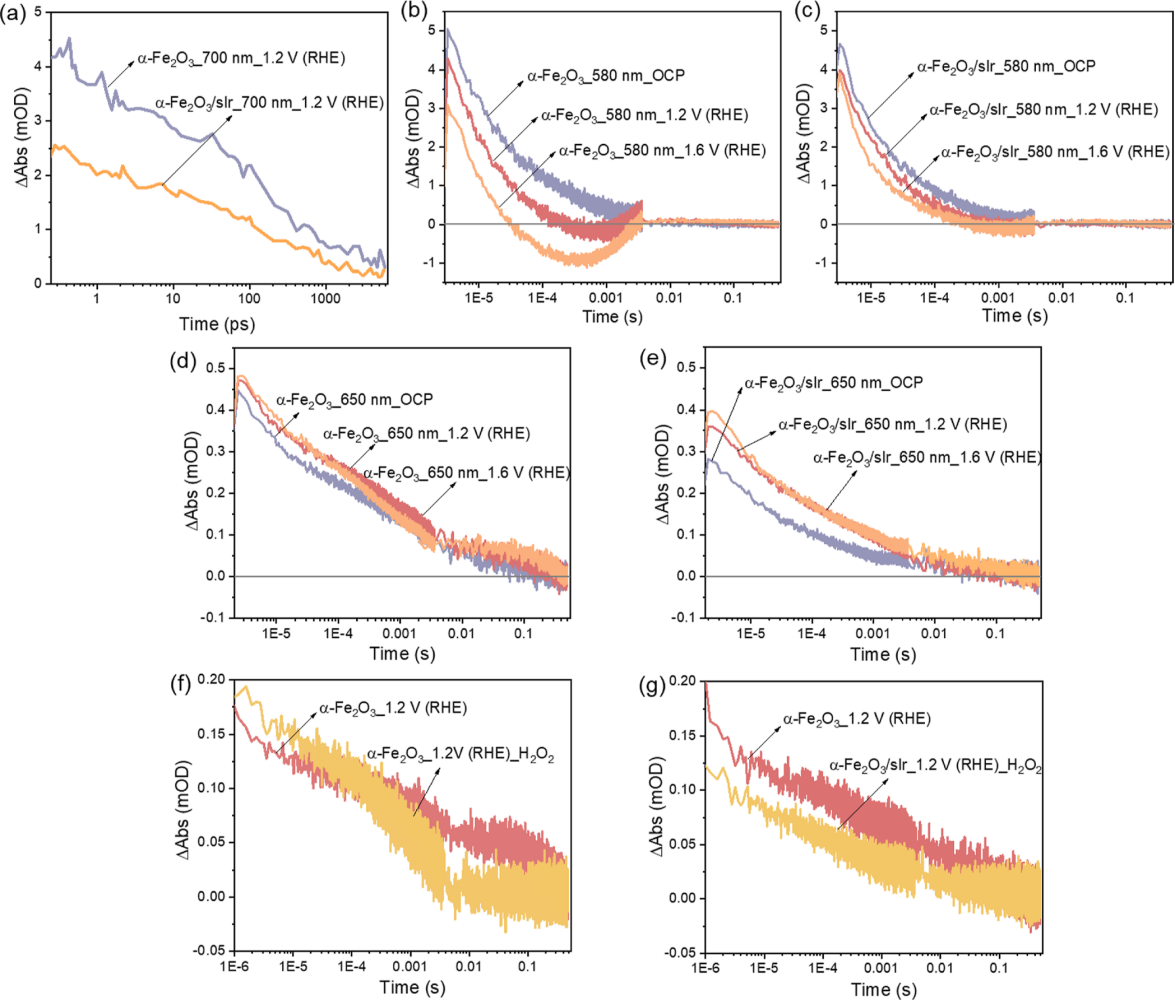
(a) Kinetic decays on the fs-to-ns timescale of α-Fe_2_O_3_ and α-Fe_2_O_3_/sIr at 700 nm at 1.2 V vs RHE; ms-to-s transient absorption kinetics probed at 580 nm of (b) α-Fe_2_O_3_ and (c) α-Fe_2_O_3_/sIr at three different potentials; ms-to-s transient absorption kinetics probed at 650 nm of (d) α-Fe_2_O_3_ and (e) α-Fe_2_O_3_/sIr at three different potentials; ms-to-s transient kinetic spectra probed at 650 nm of (f) α-Fe_2_O_3_ and (g) α-Fe_2_O_3_/sIr in the presence of H_2_O_2_ at 1.2 V vs RHE.

TAS is a well-established technique to probe the dynamics of photogenerated charge carriers in photocatalytic electrodes.^24−29^ In situ TAS was conducted on α-Fe_2_O_3_ and α-Fe_2_O_3_/sIr photoanodes at various potentials in the same cell configuration as in the PEC experiments. The obtained transient absorption spectra (Figure S7) are consistent with assignments in the literature, where 580 nm absorption has been assigned to excited-state absorption by photogenerated electrons in the conduction band (CB) of α-Fe_2_O_3_ and excitation of holes in the valence band (VB) of α-Fe_2_O_3_ to intra-band states, while the absorption at >650 nm has been assigned to intra-band hole absorption in the VB of α-Fe_2_O_3_.^25,30,31^ Absorption to oxidized Ir^4+^ can also weakly contribute to the signals at 580 nm.^32,33^ In Figure 3a, the ps-to-ns signal decay at 700 nm is plotted for α-Fe_2_O_3_ and α-Fe_2_O_3_/sIr. This decay is intensity dependent (Figure S8) due to bimolecular recombination of the photogenerated holes in the hematite.^25,30^ A reduction in the amplitude was found for α-Fe_2_O_3_/sIr relative to α-Fe_2_O_3_, in agreement with the observed difference in spectral shapes in the transient absorption spectra in Figure S7, where the ratio of the 580 to 700 nm signal increased in α-Fe_2_O_3_/sIr. This reduction could be the result of a decrease in the concentration of photogenerated holes in α-Fe_2_O_3_/sIr due to ultrafast hole transfer from hematite to sIr atoms, which was, however, not resolved in our experiments. Despite the differences in the amplitudes, a similar decay half-time (τ_1/2_) was found for α-Fe_2_O_3_/sIr (∼100 ps) and α-Fe_2_O_3_, showing that the dynamics on picosecond and nanosecond timescales are governed by charge recombination in the bulk of the hematite.

The dynamics of electrons and holes were further investigated on the microsecond to second timescales, which are more relevant to the water oxidation process.^34^Figure 3b,c shows the kinetics of α-Fe_2_O_3_ and α-Fe_2_O_3_/sIr at 580 nm under open circuit potential (OCP), 1.2 and 1.6 V versus RHE. One of the most remarkable differences between the two samples was that the decay of α-Fe_2_O_3_ was strongly dependent on the applied bias, and there was a formation of a negative signal at higher anodic biases. This result is in good agreement with previous TAS measurements of α-Fe_2_O_3_, relating the negative signal to a bleach caused by the population of electron traps thought to be oxygen vacancies close to the CB.^30^

The dynamics of the electron-trap bleach signal in α-Fe_2_O_3_ were determined by the rate of extraction of the trapped electrons to the external circuit, which subsequently controlled the rate of electron–hole recombination at the water–hematite interface.^35^ Consistent with previous studies, we observed reversal of the negative signals to positive at 0.005–0.01 s. In contrast to α-Fe_2_O_3_ (Figure 3b), the decay at 580 nm for α-Fe_2_O_3_/sIr (Figure 3c) was less sensitive to the anodic bias, and only a very weak negative signal was observed at 1.6 V versus RHE. This phenomenon can be explained by the passivation of surface electron traps in α-Fe_2_O_3_/sIr, which should lead to faster electron transport to the external circuit and a space charge layer build-up which would prevent electron–hole recombination.

Figure 3d,e shows the transient absorption dynamics of α-Fe_2_O_3_ and α-Fe_2_O_3_/sIr at 650 nm, where the signal is more closely related to photogenerated hematite holes. The decay of α-Fe_2_O_3_ and α-Fe_2_O_3_/sIr was recorded at OCP, 1.2 and 1.6 V versus RHE. Both systems exhibited bias-dependent kinetics in which a higher concentration of holes was observed at higher bias values due to a more efficient electron extraction to the external circuit, leading to reduced electron–hole recombination at the space charge layer. In α-Fe_2_O_3_, the data resolve the water oxidation on the >0.1 s timescale, whereas in α-Fe_2_O_3_/sIr, the holes are much shorter lived, and only 0.010 mOD is left at 0.1 s at 1.2 V versus RHE, which is 2.9 times smaller than the hole concentration in α-Fe_2_O_3_ on that timescale.^28,34^ The photocurrent data in Figure 2 show that α-Fe_2_O_3_/sIr outperformed α-Fe_2_O_3_, requiring considerable lower overpotentials and achieving higher photocurrents. Consequently, the shorter hole lifetimes observed in α-Fe_2_O_3_/sIr are likely due to a faster water oxidation process than in α-Fe_2_O_3_.^36^ However, it is not clear from these experiments whether hole transfer to Ir or hole transfer to water determines the observed kinetics in α-Fe_2_O_3_/sIr. To check these hypotheses, a kinetic study using H_2_O_2_ as a hole scavenger was carried out (Figure 3f,g).^37^ Even on the microsecond timescale, the amplitude of the holes in α-Fe_2_O_3_/sIr was significantly reduced in the presence of H_2_O_2_ (Figure 3g). This indicates that the extraction of holes from α-Fe_2_O_3_/sIr to the scavenger is much faster in α-Fe_2_O_3_/sIr with τ_1/2_ = 0.067 ms compared to α-Fe_2_O_3_ with τ_1/2_ = 0.36 ms (Figure 3f). This behavior further supports the conclusion that the presence of Ir accelerates the water-oxidation process.

**Figure 4:**
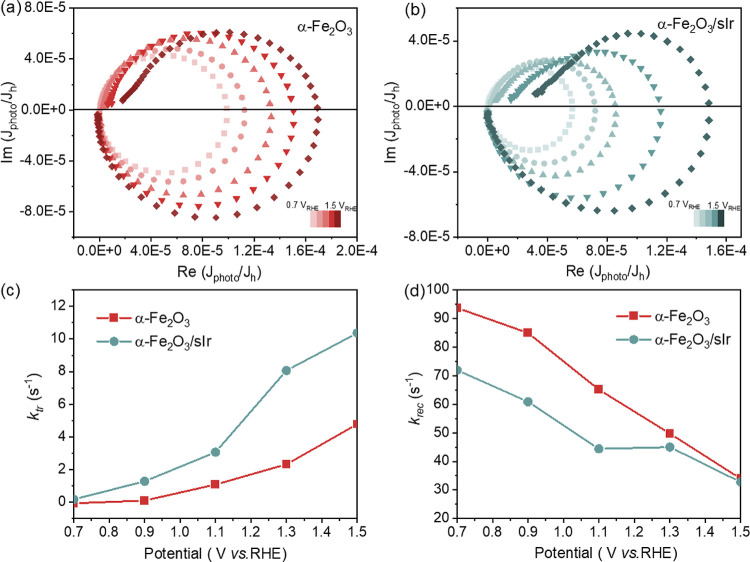
IMPS spectra of (a) α-Fe_2_O_3_ and (b) α-Fe_2_O_3_/sIr at various potentials from 0.7 to 1.5 V vs RHE; calculated (c) *K*_tr_ and (d) *K*_rec_ from IMPS spectra for α-Fe_2_O_3_ and α-Fe_2_O_3_/sIr between 0.7 and 1.5 V vs RHE.

IMPS was used to probe the surface hole transfer and recombination kinetics of hematite photoanodes.^38,39^Figure 4a,b shows the IMPS results measured between 0.7 V versus RHE and 1.5 V versus RHE for α-Fe_2_O_3_ and α-Fe_2_O_3_/sIr samples, which consist of a low-frequency semicircle in the first quadrant and a high-frequency semicircle in the fourth quadrant. The high-frequency arc in the fourth quadrant reflects the attenuation of the PEC system caused by the series resistance and capacitances, while the low-frequency arc is related to charge transfer and recombination. The hole transfer rate constants *k*_tr_ and surface recombination rate constants *k*_rec_ at the α-Fe_2_O_3_/electrolyte interface under various potentials calculated from these spectra are shown in Figure 4c,d, respectively.^40^ The values of *k*_tr_ and *k*_rec_ for α-Fe_2_O_3_ indicate an order of magnitude slower hole transfer than charge recombination in that system. *k*_tr_ gradually increases with increasing potential, while *k*_rec_ decreases. This can be explained by the fact that the applied bias contributes to extracting photogenerated electrons from the space charge layer, preventing charge recombination of electrons and holes, thus promoting hole transfer.^41^ In comparison, the α-Fe_2_O_3_/sIr sample exhibits a significantly enhanced *k*_tr_ relative to α-Fe_2_O_3_ within the whole measured potential range. For example, the *k*_tr_ value of α-Fe_2_O_3_/sIr is three times that of α-Fe_2_O_3_ at 1.3 V versus RHE. The larger *k*_tr_ of α-Fe_2_O_3_/sIr indicates a fast hole transfer,^41^ in good agreement with the TAS results. These findings further reveal that the improved PEC performance for α-Fe_2_O_3_/sIr is primarily due to the faster water oxidation rate in the presence of sIr and slower electron–hole recombination rate.

### Band Structure of α-Fe_2_O_3_/sIr

**Figure 5:**
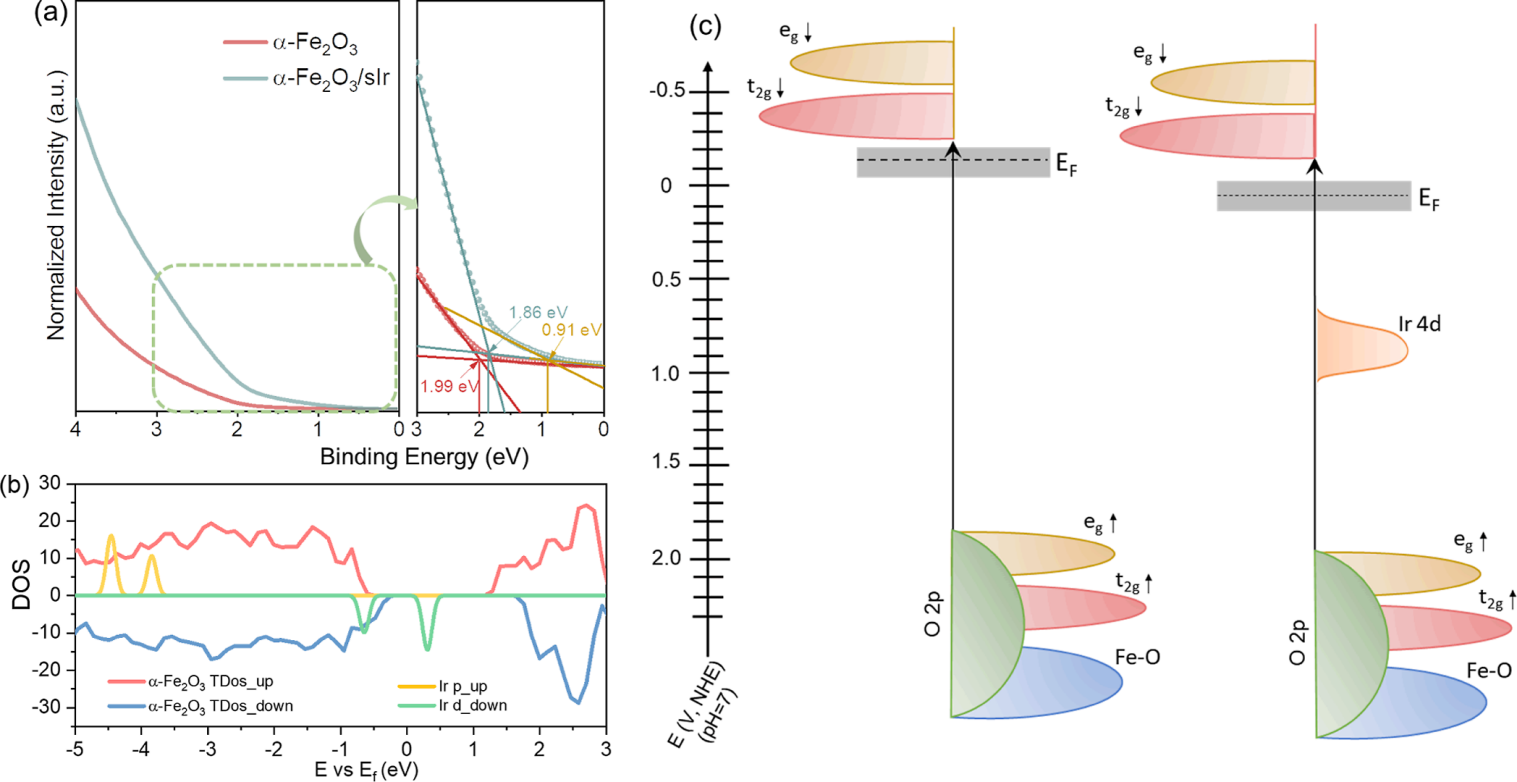
(a) Ultraviolet photoelectron spectra of α-Fe_2_O_3_ and α-Fe_2_O_3_/sIr, (b) DFT-computed PDOS of the bulk structures of α-Fe_2_O_3_ and Ir atom, and (c) energy band diagrams of α-Fe_2_O_3_ and α-Fe_2_O_3_/sIr.

To unravel the reason leading to the fast hole transfer in the presence of sIr, as demonstrated by TAS and IMPS measurements, the band structures of α-Fe_2_O_3_ and α-Fe_2_O_3_/sIr were analyzed. Figure 5a shows the ultraviolet photoelectron spectra of α-Fe_2_O_3_ and α-Fe_2_O_3_/sIr, which provide the valence structure of the measured samples. In the presence of sIr, the Fermi level relative to the VB of α-Fe_2_O_3_ is lowered by 0.13 eV. Most strikingly, a mid-gap sitting 0.91 eV below the Fermi level is found for α-Fe_2_O_3_/sIr, which is likely caused by the sIr 4d orbital energy level. The projected density of states (PDOS) of the bulk structures of α-Fe_2_O_3_ and Ir atom obtained with DFT confirms this interpretation (Figure 5b), which suggests the existence of sIr 4d states at 0.9 eV below the Fermi level and in the mid-gap of the band structure of α-Fe_2_O_3_. To draw a full picture of their band structures, UV–vis DRS and Mott–Schottky measurements were further conducted. Figure S9a shows the UV–vis results of α-Fe_2_O_3_ and α-Fe_2_O_3_/sIr, from the Tauc plot (inset of Figure S9a) of which the band gap is estimated to be 2.08 eV. From the intercepts of the linear fitting of the Mott–Schottky plots (Figure S9b), the flat band potentials for α-Fe_2_O_3_ and α-Fe_2_O_3_/sIr are determined to be 0.26 V and 0.36 V versus RHE, respectively. Generally, the CB potential of n-type semiconductors is 0.1–0.2 V higher than that of the flat band potential.^42^ Taking 0.1 V as the potential difference, the CB potentials for α-Fe_2_O_3_ and α-Fe_2_O_3_/sIr are 0.16 and 0.26 V versus RHE, respectively, corresponding to −0.25 and −0.15 V versus NHE (pH = 7). By subtracting CB potentials from band gap energy, the VB potentials of α-Fe_2_O_3_ and α-Fe_2_O_3_/sIr can be obtained. Combining the above information, the energy band diagrams of α-Fe_2_O_3_ and α-Fe_2_O_3_/sIr are presented in Figure 5c.^43,44^ The sIr 4d mid-gap energy level could serve as a hole-trap center, therefore leading to the fast hole transfer from α-Fe_2_O_3_ to sIr.

### DFT Calculation of Water Oxidation Mechanism of α-Fe_2_O_3_/sIr

**Figure 6:**
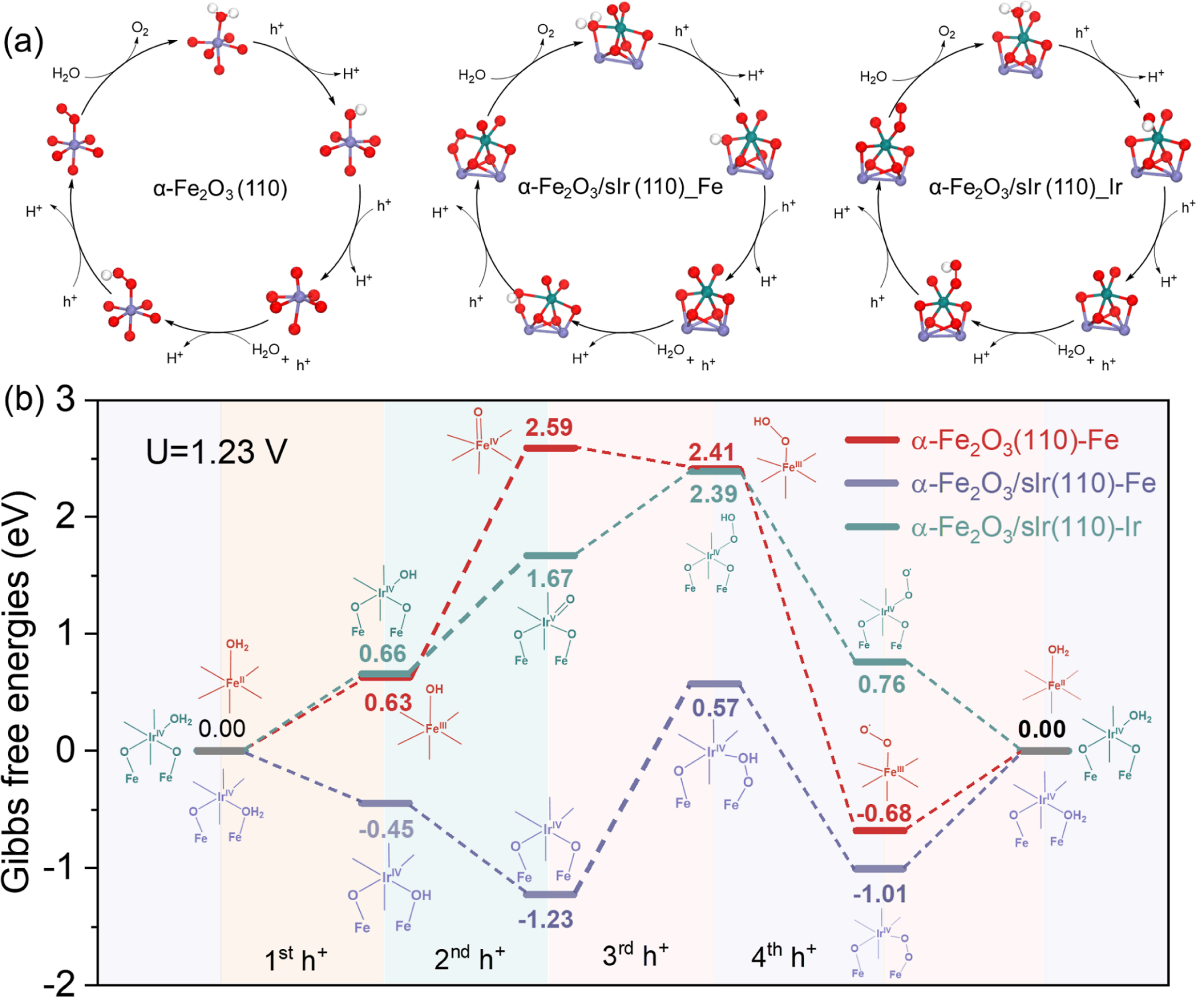
(a) Proposed mechanism and optimized structures of the reaction intermediates of the oxygen evolution reaction on α-Fe_2_O_3_ (110) (left), on the Fe site of α-Fe_2_O_3_/sIr (110) (middle), and on the Ir site of α-Fe_2_O_3_/sIr (110) (right). The red balls are O atoms, the Nile blue balls are Ir atoms, the purple balls are Fe atoms, and the white balls are H atoms; (b) Gibbs free energy diagram depicting the three reaction mechanisms considered in the DFT calculations. The reaction profiles on α-Fe_2_O_3_ (110) are displayed with red; the reaction mechanism of α-Fe_2_O_3_/sIr (110) with Fe as the active site is shown with purple and that with Ir as a sole catalytic site is shown with Nile blue.

Using DFT calculations, we investigated the water oxidation on the (110) surfaces of α-Fe_2_O_3_ and α-Fe_2_O_3_/sIr, one of the most dominating facets of hematite synthesized in this work. We modeled the water oxidation mechanism on α-Fe_2_O_3_ (110) and α-Fe_2_O_3_/sIr (110), as shown in Figure 6a. For α-Fe_2_O_3_/sIr (110), two possible active sites were considered: Ir or Fe. The details of the atomistic surface models and DFT calculations are reported in the Supporting Information (Figures S10 and S11). Figure 6b shows the Gibbs free energies at the equilibrium potential of 1.23 V of the intermediates involved in the water oxidation reaction. On α-Fe_2_O_3_ (110), after the water adsorption on the Fe center, four consecutive proton-electron transfer processes generate the surface-trapped holes −Fe^IV^=O and −Fe^III^–OH, the O–O (H) bond, and the surface superoxide species, subsequently released as O_2_. This DFT computed pathway agrees well with the experimentally demonstrated water oxidation mechanism on hematite photoanodes.^45^ The second hole transfer and oxidation of Fe^III^–OH to Fe^IV^=O on α-Fe_2_O_3_ (110) requires a free energy of 1.96 eV, which is the limiting reaction barrier.^46^

On hematite, the large overpotential leads to fast electron–hole recombination, which explains the inefficient water oxidation observed experimentally. In the case of α-Fe_2_O_3_/sIr (110), when still considering Fe as the active site, the limiting step is the third-hole oxidation of O* to OOH* with the limiting energy barriers also as large as 1.80 eV. Thus, the water oxidation on the Fe site of α-Fe_2_O_3_/sIr (110) is not consistent with the fast hole transfer and water oxidation kinetics observed experimentally. The final mechanism considered is with Ir as the active site, shown with a Nile blue color in Figure 6b. This mechanism is the most efficient in promoting the reaction, as the energy barrier of the limiting reaction step, the second oxidation of Ir^IV^–OH to Ir^IV^=O, is only 1.01 eV. The Gibbs free energy adsorption of intermediates (see Table S2) and Bader charge analysis were further adopted to rationalize the lower energy barrier of water oxidation on α-Fe_2_O_3_/sIr (110) with Ir as the active site. The Gibbs free energy adsorption values shown in Table S2 suggest a relatively weaker coupling of oxygen-containing intermediates with the Ir of α-Fe_2_O_3_/sIr (110), corresponding to the comparatively more positive adsorption energy (see Table S2), which lowers the energy barrier for the reaction to proceed.^46^ This is further supported by the Bader charge analysis, which indicates a reduced charge transfer between the intermediates (O, OOH, and OO) and the Ir (Figure S12b) as compared to the charge transfer between these intermediates and the Fe of α-Fe_2_O_3_/sIr (110) (Figure S12a). Therefore, our calculations indicate that sIr acts as the active site for the reaction, behaving as a true catalyst on hematite photoanodes, promoting the hole transfer, and accelerating the water splitting reaction, which is in line with the TAS and IMPS experiments.

## Conclusions

In this work, we explored the role of Ir loaded on α-Fe_2_O_3_ as a cocatalyst in the water splitting mechanism. Our combined investigation using in situ TAS, IMPS, and DFT calculations showed that sIr acts as a true catalyst, accelerating the solar water oxidation reaction. The TAS experiments indicate a reduced hole concentration and a shortened lifetime of the holes for hematite in the presence of sIr due to a faster hole transfer process. The IMPS data also support the improved hole-transfer rates in α-Fe_2_O_3_/sIr. Our energy band structure calculations of α-Fe_2_O_3_ and α-Fe_2_O_3_/sIr showed that sIr induces mid-gap states with Ir 4d orbitals, which could serve as hole traps, facilitating the hole transfer from α-Fe_2_O_3_ to sIr followed by fast water oxidation. Our DFT calculations confirmed that the most favorable water oxidation pathway in α-Fe_2_O_3_/sIr involves sIr as the active site instead of Fe. The reaction on the sIr site has a significantly lower energy barrier (1.01 eV) than when Fe acts as the active site (1.80 eV). Consequently, Ir acts as a true catalyst, accelerating the water oxidation steps rather than just extending the lifetime of photogenerated holes. These results provide for the first time a deeper understanding of the interplay between the electronic structure, hole transfer, and depletion in water oxidation mechanisms. More broadly, our investigation indicates that the creation of hole trap states involving the single atom can be used as a design principle for engineering efficient single-atom cocatalysts on photoanodes.

## Supporting Information Available

Experimental and DFT computational details; XPS data and analysis of mIr on FTO before and after PCT with different durations; additional HAADF-STEM images of α-Fe_2_O_3_/sIr; crystal lattice analysis of the synthesized α-Fe_2_O_3_ from HAADF-STEM images; XPS survey spectrum of α-Fe_2_O_3_, α-Fe_2_O_3_/mIr, and α-Fe_2_O_3_/sIr; relative element percentages (at. %) of α-Fe_2_O_3_, α-Fe_2_O_3_/mIr, and α-Fe_2_O_3_/sIr from XPS spectra; high-resolution XPS spectra of C 1s for α-Fe_2_O_3_, α-Fe_2_O_3_/mIr, and α-Fe_2_O_3_/sIr; fs-to-ns and ms-to-s TAS absorption spectra of α-Fe_2_O_3_ and α-Fe_2_O_3_/sIr; fs-to-ns TAS kinetic profiles at different pump intensities; UV–vis spectra and Mott–Schottky plots of α-Fe_2_O_3_ and α-Fe_2_O_3_/sIr; DFT models of binding sites and binding energy for single Ir metal atoms on a α-Fe_2_O_3_ (110) surface; DFT models for the structure of the hydroxyl terminated α-Fe_2_O_3_ (110) surface and α-Fe_2_O_3_/sIr (110) surface; adsorption Gibbs free energies of important intermediates on α-Fe_2_O_3_ (110) and α-Fe_2_O_3_/sIr (110); and Bader charge analysis of the metal center and intermediates involved in the water oxidation reaction on α-Fe_2_O_3_/sIr (110)_Fe and α-Fe_2_O_3_/sIr (110)_Ir (PDF)
