# Supplementary material for: Single-Atom Iridium on Hematite Photoanodes for Solar Water Splitting: Catalyst or Spectator?
Source: J Am Chem Soc. 2023 Jan 11;145(3):1686–95. doi: 10.1021/jacs.2c09974 (PMC9880996; doi:10.1021/jacs.2c09974)
Supplement: Supplementary file 1 — ja2c09974_si_001.pdf [file ja2c09974_si_001.pdf]

Supporting Information

Single-atom Iridium on Hematite Photoanodes for Solar Water Splitting: Catalyst or Spectator?

Qian Guo[1], Qi Zhao[2], Rachel Crespo-Otero[2], Devis Di Tommaso[2], Junwang Tang[3], Stoichko D. Dimitrov[2], Maria-Magdalena Titirici[4], Xuanhua Li[5], Ana Belén Jorge Sobrido[1]*

[1]School of Engineering and Materials Science, Queen Mary University of London, E1 4NS, London, UK

[2]School of Physical and Chemical Sciences, Queen Mary University of London, E1 4NS, London, UK

[3]Department of Chemical Engineering, University College London, Torrington Place, WC1E 7JE, London, UK

[4]Department of Chemical Engineering, Imperial College London, SW7 2AZ, London, UK

[5]State Key Laboratory of Solidification Processing, Center for Nano Energy Materials, School of Materials Science and Engineering, Northwestern Polytechnical University, Xi'an 710072, China.

Table of Contents

Methods 15

XPS data and analysis of mIr on FTO before and after PCT with different durations 18

Additional HAADF-STEM images of α-Fe_2O_3/sIr 20

Crystal lattice analysis of the synthesized α-Fe_2O_3 from HAADF-STEM images........................22

XPS survey spectrum of α-Fe_2O_3, α-Fe_2O_3/mIr, and α-Fe_2O_3/sIr 23

Relative element percentages (at%) of α-Fe_2O_3, α-Fe_2O_3/mIr, and α-Fe_2O_3/sIr from XPS spectra23

High-resolution XPS spectra of C1s for α-Fe_2O_3, α-Fe_2O_3/mIr, and α-Fe_2O_3/sIr..........................24

fs-ns and ms-s TAS absorption spectra of α-Fe_2O_3 and α-Fe_2O_3/sIr 25

fs-ns TAS kinetic profiles probed at 740 nm at different pump intensities 25

UV-Vis spectra and Mott-Schottky plots of α-Fe_2O_3 and α-Fe_2O_3/sIr..26

DFT models of binding sites and binding energy for single Ir metal atoms on a α-Fe_2O_3 (110) surface 26

DFT models for the structure of the hydroxyl terminated α-Fe_2O_3 (110) surface and α-Fe_2O_3/sIr (110) surface...27

Adsorption gibbs free energies of important intermediates on α-Fe_2O_3 (110) and α-Fe_2O_3/sIr (110) 27

Bader charge analysis of metal centre and intermediates involved in the water oxidation reaction on α-Fe_2O_3/sIr (110)_Fe and α-Fe_2O_3/sIr (110)_Ir 29

References 29

Methods

*Synthesis of hematite (*α-Fe_2O_3*) photoanodes*

A piece of well cleaned fluorine-doped tin oxide glass slide (FTO, SnO_2/F, ~13 Ω/sq, Sigma-Aldrich Co.) by successive sonication in acetone, isopropanol, and deionized water for 15 min, respectively, was put into an autoclave containing 0.15 M $FeCl_3$ ($FeCl_3 \cdot 6H_2O$, ≥ 99.0%, Sigma-Aldrich Co.) and 1 M $NaNO_3$ (≥ 99.0%, Sigma-Aldrich Co.) solution, followed by reaction at 100 ^oC for 1 h. The obtained uniform yellow layer of iron oxyhydroxides (β-FeOOH) film on FTO was converted into red hematite (α-Fe_2O_3) by annealing at 800 ^oC for 5 min after thoroughly rinsed with deionized water. Note that the backside of FTO was covered with Kapton tape during the reaction, preventing back cover of FeOOH, which was completely removed before calcination. In order to achieve optimum performance, the process was repeated one more time to obtain the final α-Fe_2O_3 photoanodes.[1] Deionized water (~18.25 MΩcm, 25 °C) produced by a Milli-Q Element System (Millipore, Molsheim, France) was used all through the experiments.

*Synthesis of single atom iridium on hematite (*α-Fe_2O_3*/sIr)*

Single-atom iridium (sIr) decorated hematite photoanode (α-Fe_2O_3/sIr) was synthesized following a previously reported photochemical approach.[2] The as-prepared α-Fe_2O_3 was firstly immersed overnight into a 0.5 mM of molecular iridium precursor (mIr) solution ([2-(pyridine-2yl)-2-propanato]iridium(IV) dimer solution 97%, 1 mM in 0.1 M aqueous $NaIO_3$ solution, Strem Chemicals, Inc.), followed by washed thoroughly with deionized water and carefully dried under flowing nitrogen. After this heterogenization process, a layer of mIr was bonded on hematite surface.[3] The obtained sample was denoted as α-Fe_2O_3/mIr. The α-Fe_2O_3/mIr was then subjected to a photochemical treatment for 25 min under UV light in a UV Ozone cleaner chamber (ProCleaner™ Plus, BioForce Nanosciences, Ames, USA), during which process the organic ligands of mIr were completely decomposed, finally leading to single Ir atoms on hematite (α-Fe_2O_3/sIr).

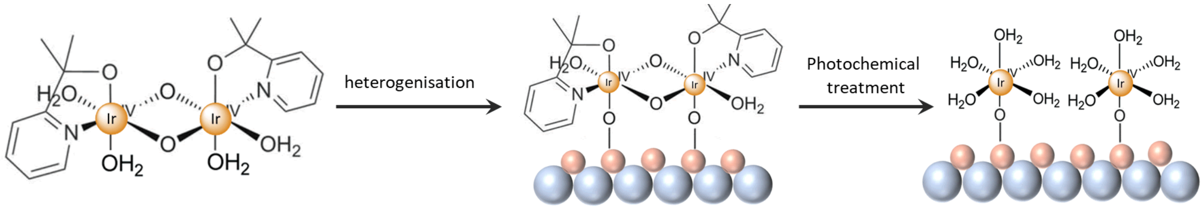

Scheme S1. Schematic illustration of the synthesis procedure for α-Fe_2O_3/sIr (Blue ball is O atoms, pink ball is Fe atoms).

Optical, structural and morphological characterization

The morphology and thickness of the as-prepared hematite films was observed by field emission scanning electron microscopy (FE-SEM, JEOL JSM-6301F) at a beam voltage of 5 keV and using a secondary electron detector. No conductive coating was used on the samples. Isolated sIr states on hematite were assessed by a double-corrected microscope dedicated to atomic scale imaging (JEOL ARM300CF) at the electron Physical Science Imaging Centre (ePSIC) in Diamond Light Source. High-angle annular dark-

field imaging- scanning transmission electron microscope (HAADF-STEM) images were collected at an operation voltage of 300 kV. Samples for the STEM characterization were prepared by directly scratching the as-prepared α-Fe_2O_3 and α-Fe_2O_3/sIr samples on a lacey caron Cu grid.[4] Images were analysed by Gatan Digital Micrograph software. X-ray diffraction (XRD) patterns were performed with a Panalytical Xpert Pro diffractometer with Cu Kα radiation (1.5418 Å). UV-vis absorption spectra of all photoanodes were recorded with a UV-vis spectrophotometer (Lambda 950, Perkin Elmer) equipped with an integrating sphere (150 mm diameter sphere covered with Spectralon as the reflecting material, Perkin Elmer) within a wavelength range of 350 to 800 nm and a step of 1 nm. X-ray photoelectron spectroscopy (XPS) analysis was carried out on a Thermo ScientificNEXSAXPS system with an Al Kα X-ray source, and the data was analysed by Thermo Avantage software. Ultraviolet photoelectron spectroscopy (UPS) was also conducted on the Thermo ScientificNEXSAXPS system using He I radiation.

PEC characterization

Photocurrent measurements were carried out in a three-electrode set up with α-Fe_2O_3 or α-Fe_2O_3/sIr of 1 cm^2 as the working electrode, Ag/AgCl (KCl sat.) as the reference electrode, and a platinum plate as counter electrode in 0.1 M KNO_3 aqueous electrolyte (pH 7.0).A computer-controlled potentiostat (Gamry Instrument Interface 5000E) was used to apply bias on the three-electrode system. A LED light source with wavelength of 480 nm (Zahner CIMPS System) was used to excite the photoelectrodes from the electrode/electrolyte site. Linear sweep voltammetry (LSV) measurements were conducted at a scan rate of 20 mV/s in the voltage range of 0.7 V–1.6 V *vs.* RHE.Intensity modulated photocurrent spectroscopy (IMPS) analysis was carried out using a potentiostat (IVIUM technology) under modulated illumination (37.5 $mWcm^{-2}$) of 450 nm LED (ModuLightmodule, IVIUM technology) in the same set up as that for LSV tests from 0.7 V to 1.5 V *vs.* RHE with a step of 0.2 V. A modulation of 10% in light intensity was applied, over a frequency range from 10 kHz to 0.1 Hz at each potential step. IMPS spectra were fitted using Zview software (Scribner).

Transient absorption spectroscopy characterization

Transient absorption spectroscopy (TAS) was carried out with two separate instrument. Picsecond-nanosecond TAS was carried out using a Helios spectrometer (Ultrafast systems) seeded by a Ti:Sapphire regenerative amplifier (Solstice by MKS Spectra-Physics) with an 800 nm, 1 KHz pulses. The pump pulse was generated by a TOPAS-NIRUVIS (Light Conversion). The experiments were conducted in-situ on complete cells as in the photoelectrochemical experiments conducted in this study using varied pump fluence. Sample degradation was not observed during experiments. The microsecond-second TAS was conducted using the 355 nm pulse light generated by a Nd:YAG OPOTEK Opolette 355 II optical parameteric oscillator. The experiments were carried out with the fluence of 400 $\mu J/cm^2$ and at the laser repetition rate of 0.8 Hz. Probe light was generated by a 100 W Bentham IL1 quartz halogen lamp and send through ND and coloured filters and a monochromator to remove scattered light and record single wavelength kinetics. A Costronics pre-amplification detection system was connected to a Tektronics oscilloscope a National Instrument DAQ card. Data was acquired and processed using software written in the LabVIEW environment (Austin Consultants). Data analysis was carried our with Origin. Care was

taken to avoid sample degradation, and when such was observed upon application of high anodic bias, the sample was replaced.

Density Functional Theory (DFT) Calculations

All the spin-polarized DFT calculations in this work were conducted using the Vienna *ab initio* simulation package (VASP)[5] with a plane-wave cut-off of 450 eV and a Monkhorst-Pack *k*-point samplings of (3×3×1). To accurately treat the Fe d-electron correlation, DFT+U calculations with a value of Ueff = 4.0 eV was applied to both the Fe d states and Ir d states, using the formalism suggested by Dudarev *et al.*.[6] Starting from the fully relaxed bulk structures, the *p*(2×2) supercell slab was used to model the α-Fe_2O_3 (001) and the unit cell was used for α-Fe_2O_3 (110).

Based on previous study, a fully hydroxylated surface was used to study solar water splitting mechanism.[2] Projector augmented wave (PAW) method[7,8] together with the PBE exchange-correlation functional[9] were employed to describe the electron-ion interactions. The Grimme's -D3 dispersion correction was performed to provide a more accurate description of the dispersion interactions.[7,9] The energy convergence criterion was set to be 10^{-6} eV per unit cell and the geometry convergence criterion was set to be 1×10^{-2} eV per unit cell for energy difference between two consecutive ionic steps. During this calculation, the bottom two layers were fixed and the remaining layers including the adsorbed species were relaxed. A vacuum gap spacing of 20 Å was used to prevent self-interaction. The calculations of the energy for catalytic steps were performed without considering solvation effects.Vaspkit[10] was used to calculate thermal corrections to the Gibbs free energies. For reaction steps that involved H^+ and e^-, the computational hydrogen electrode model developed by Nørskov *et al.*[11,12] was used to calculate the free energy changes. The Gibbs free energy analysis was performed under standard conditions (pH = 0, 298.15K, 1 atm) and U= 1.23 V.

XPS data and analysis of mIr on FTO before and after PCT with different durations

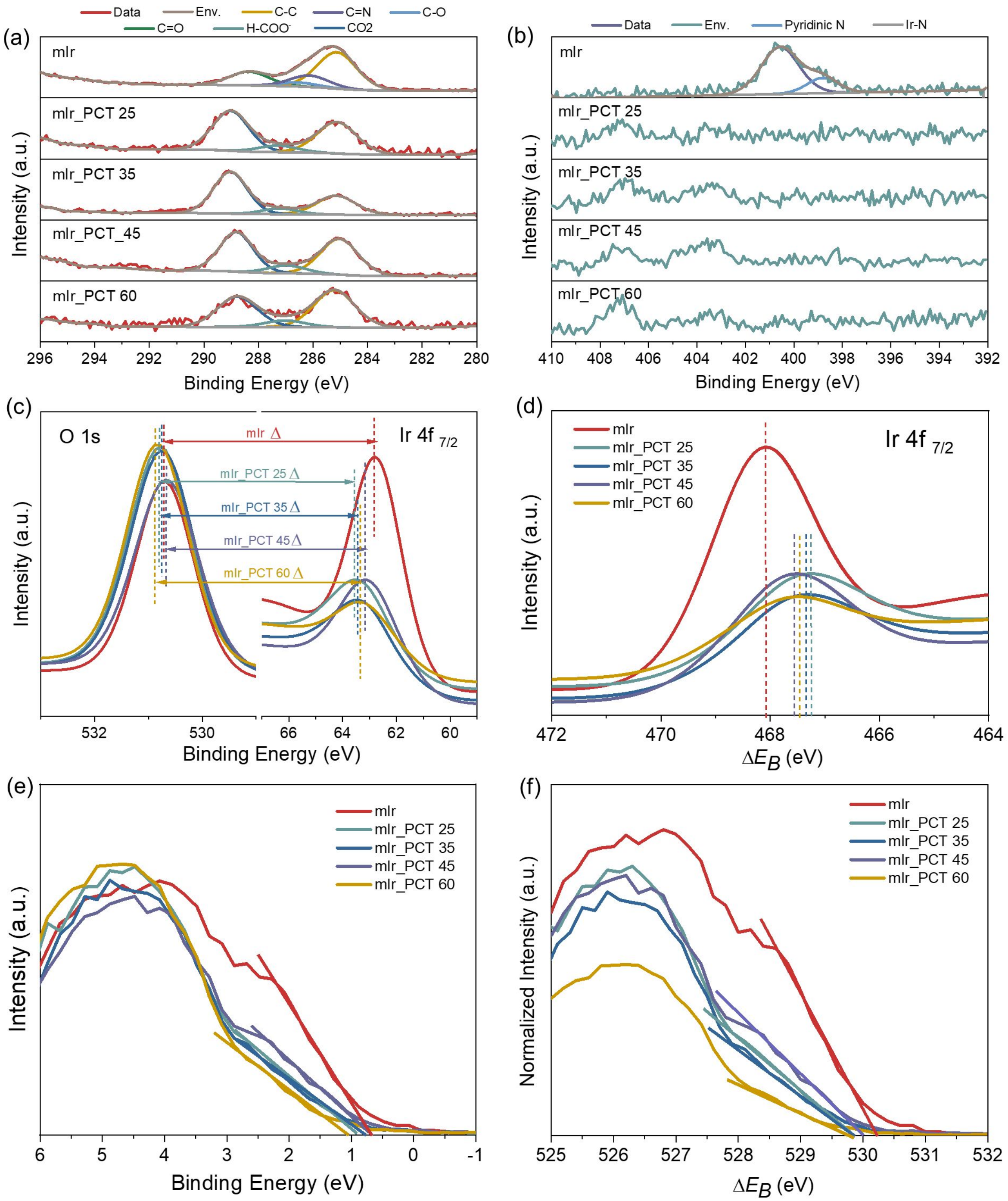

Figure S1. XPS results of mIr on FTO substrates before and after PCT of 25 min (mIr_PCT 25), 35 min (mIr_PCT 35), 45 min (mIr_PCT 45), and 60 min (mIr_PCT 60). (a) XPS C 1s spectra; (b) XPS N 1s spectra; (c) XPS O^{2-} (oxide) component of O 1s spectra (left) and XPS Ir $4f_{7/2}$ spectra (right); (d) the alignment of Ir $4f_{7/2}$ component spectra plotted on the ΔE_B scale referenced against the O^{2-}; (e) XPS VB spectra; and (f) VB spectra on the ΔE_B scale referenced against the O^{2-}.

Before employing this approach to the synthesis of α-Fe_2O_3/sIr, we firstly explored the effects of the PCT duration on getting Ir atoms from its molecular precursor. Therefore, mIr loaded on FTO substrates were prepared followed by the photochemical treatment for durations of 25 min, 35 min, 45 min, and 60 min. The obtained samples are denoted as mIr_PCT 25; mIr_PCT 35, mIr_PCT 45, and mIr_PCT 60, correspondingly. Starting from PCT of 25 min is based on the previously reported work.[2] XPS measurements on these samples were conducted. As shown in Figure S1a, the C 1s spectra of mIr can be further deconvoluted into C-C (sp2 and sp3), C=N, C-O, and C=O, correlating well with the chemical environment of C in mIr. After 25 min PCT, the C 1s spectrum is deconvoluted into C-C (sp^2, 285.2 eV), H-COO^-(287.3 eV), CO_2 (288.8 eV), with absence of C=N, suggesting the decomposition of some of the organic ligands.[13] Longer PCT (from 35 min to 60 min), results into no significant changes in the C 1s spectrum, indicating that the decomposition of organic ligands has been mostly completed after 25 min of PCT. Figure S1b shows the N 1s spectra of mIr before and after PCT. A significant N 1s peak is observed for mIr, which can be further deconvoluted into pyridinic N and Ir-N, in good agreements with the N environment in mIr. However, after PCT, the N 1s peak disappears accompanied by the new appearance of some NO_x peaks, evidencing the complete decomposition of the mIr ligands during the PCT process. Furthermore, we explored the chemical state of Ir in mIr before and after the PCT procedure. As shown in Figure S1c, an upshift of Ir $4f_{7/2}$ is found for mIr_PCT samples as compared to the mIr sample. However, shifts of O 1s peaks mainly corresponding to O^{2-} (oxide) component are also observed among these samples. Therefore, to preclude the misalignment of the binding energy scale caused by the shift of Fermi level position, the separation of the O^{2-} and Ir $4f_{7/2}$ peaks (indicated by Δ, Figure S1c) is employed here to evaluate the real chemical shift of Ir $4f_{7/2}$. For this, Ir $4f_{7/2}$ peaks of each sample were replotted in Figure S1d on the ΔE_B scale referenced against the O^{2-} peak energy.[14] This allows a direct comparison of the energies of Ir states. Note that a higher ΔE_B corresponds to a lower binding energy and vice versa. The Ir 4f $_{7/2}$ peaks for mIr_PCT samples situates at a similar position on the ΔE_B scale (around 467.3 eV), which is 0.8 eV lower than that of the mIr sample (468.1 eV), indicating a higher oxidation states of Ir after the PCT process as a result of the loss of electron donor ligands. Valence spectra (Figure S1e) and valence spectra on the ΔE_B scale referenced against the O 1s peak energy (Figure S1f) of these samples were also analyzed to evaluate the change of their electronic structure. The energies of their valence band level were obtained by extrapolating the first linear part of the valence spectra. Figure S1e and S1f indicate an upshift of the valence band level corresponding to the 4d orbital energy level of Ir after the PCT process. As shown in Figure S1f, the VB of mIr_PCT samples sits at around 529.9 eV on the ΔE_B scale, which is 0.3 eV below that of the mIr sample. These results provide additional evidence for the complete decomposition of ligands.

Additional HAADF-STEM images of α-Fe_2O_3/sIr

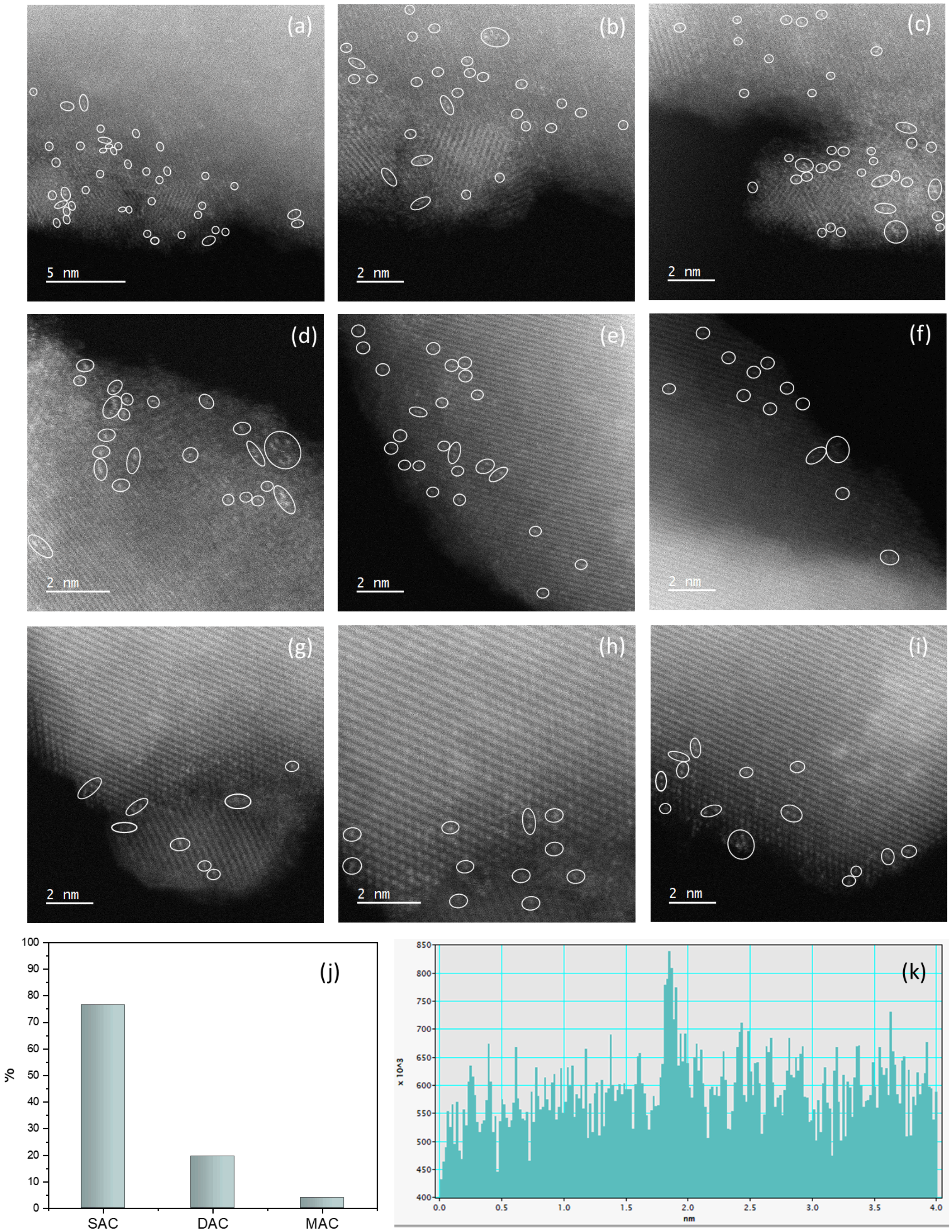

Figure S2. (a–i) More recorded data of Ir SAC on hematite; (j) Ir SAC percentage of detected Ir atoms on α-Fe_2O_3 surface; (k) HAADF intensity profile taken along the atoms on α-Fe_2O_3/sIr samples.

More data of recorded Ir SAC on hematite by HAADF-STEM are provided in Figure S2. In these images, approximately 200 of Ir units were observed. Among them, 153 of Ir SAC, 39 of Ir diatom catalyst (DAC), and 8 of Ir cluster (atom ≥ 3) were recorded at the atomic resolution. The Ir SAC takes up 79% of all observed Ir units, as shown in Figure S2, evidencing most Ir are present as single atoms on the hematite surface. The HAADF intensity line profile in Figure S2 was taken along the atoms. The sharp bump in the HAADF profile is assigned to the Ir atom, while the rest shorter peaks are due to Fe atoms. The isolate Ir peak in the HAADF intensity profile further confirms the existence of a single Ir atom with a diameter of *ca.* 0.1 nm on the surface of hematite support.

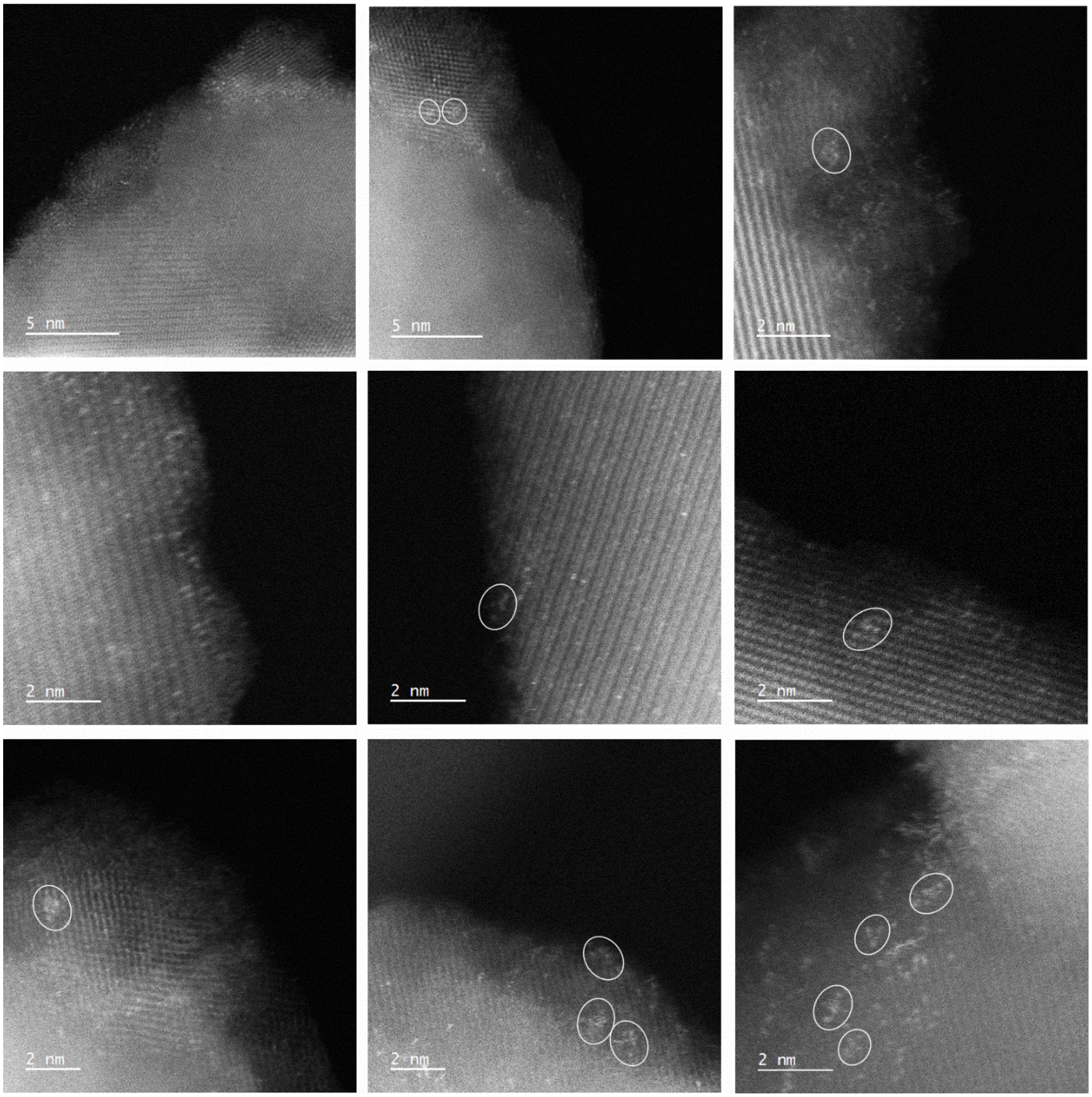

Figure S3. HAADF-STEM images of α-Fe_2O_3/sIr prepared by PCT of α-Fe_2O_3/mIr for 35 min.

Exposing the α-Fe_2O_3/mIr for a longer PCT duration of 35 min to prepare α-Fe_2O_3/sIr led to several more aggregates of Ir atoms, as shown in Figure S3, where the Ir clusters marked by a white circle. Therefore, 25 min was selected as the optimum length of time for the PCT.

Crystal lattice analysis of the synthesized α-Fe_2O_3 from HAADF-STEM images

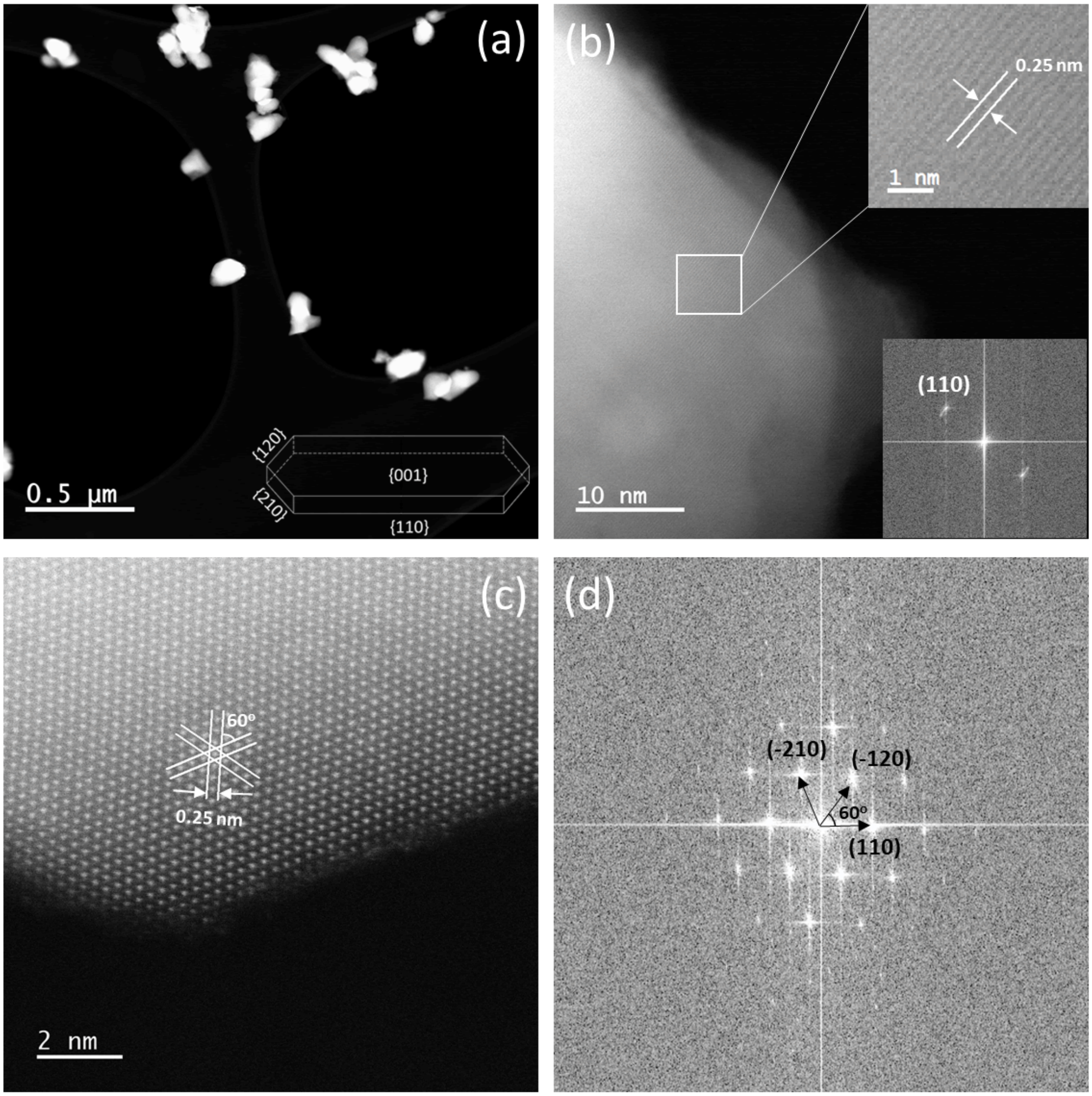

Figure S4. (a) HAADF-STEM image of hematite nanocrystals (inset: geometrical model of a hematite nanorod with exposed facets); (b) HAADF-STEM image on a single hematite nanocrystal and the corresponding FFT pattern (inset) showing (110) indices; (c) A highly magnified HAADF-STEM image on a single hematite nanocrystal having lattice planes with (110), (−120), and (−210) indices and the corresponding FFT pattern has lattice spots with (110), (−120), and (−210) indices (d).

Figure S4a shows that the synthesized hematite crystals resembles a short-nanorod shape. It has been reported that (001) and (110) facets are the dominant facets for hematite nanorods (inset of Figure S4a).[15-17] The HAADF-STEM image (Figure S4b) taken on an individual crystal and its fast Fourier transforms (FFT; inset in Figure S4b) indicates that the synthesized hematite with (110) lattice fringes (0.25 nm). A highly magnified image of the individual crystal (Figure S4c) and its corresponding FFT pattern (Figure S4d) resolves three sets of lattice fringes. Their lattice spacing is calculated to be 0.25 nm, and the interfacial angle between them is found to be 60°, fitting well to the (110), (-120), and (-210) planes of hematite, respectively. These observations are in good agreement with those reported for hematite crystals with (110) facet as one of the dominant facets in the literature.[15-17]

XPS survey spectrum of α-Fe_2O_3, α-Fe_2O_3/mIr, and α-Fe_2O_3/sIr

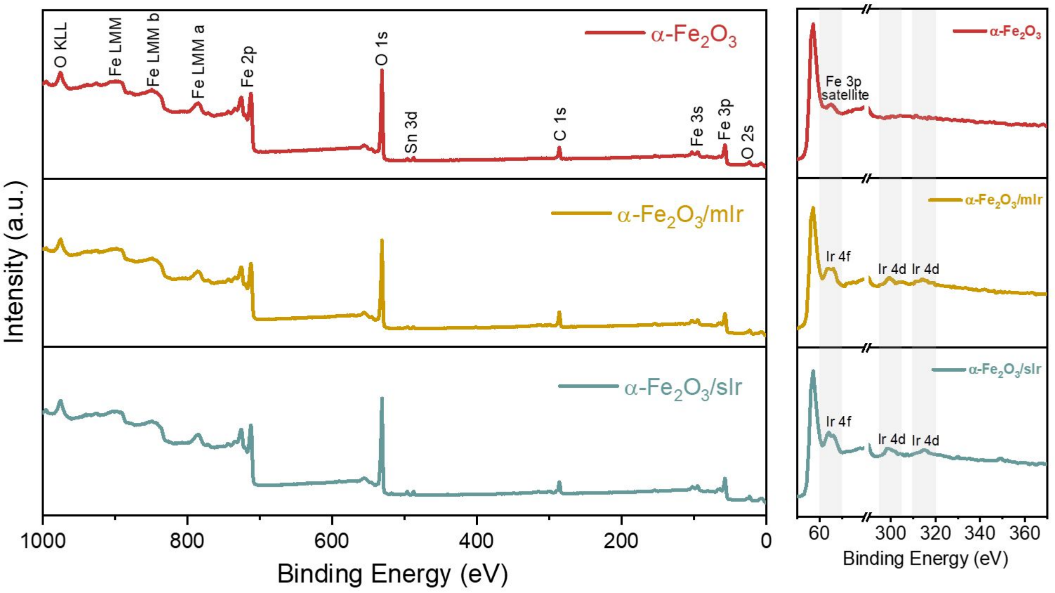

Figure S5. XPS survey spectrum of α-Fe_2O_3, α-Fe_2O_3/mIr, and α-Fe_2O_3/sIr.

Relative element percentages (at%) of α-Fe_2O_3, α-Fe_2O_3/mIr, and α-Fe_2O_3/sIr from XPS spectra

Table S1. The relative element percentages (at%) of each sample from XPS spectra.

	α-Fe_2O_3	α-Fe_2O_3/mIr	α-Fe_2O_3/sIr

C 1s	21.95 %	24.05 %	20.47 %
N 1s	--	1.46 %	--
O 1s	58.65 %	56.99 %	61.0 %
Fe 2P	19.40 %	17.12 %	18.13 %
Ir 4f	--	0.38 %	0.41 %
Ir/Fe	--	2.22 %	2.26 %

High-resolution XPS spectra of C1s for α-Fe_2O_3, α-Fe_2O_3/mIr, and α-Fe_2O_3/sIr

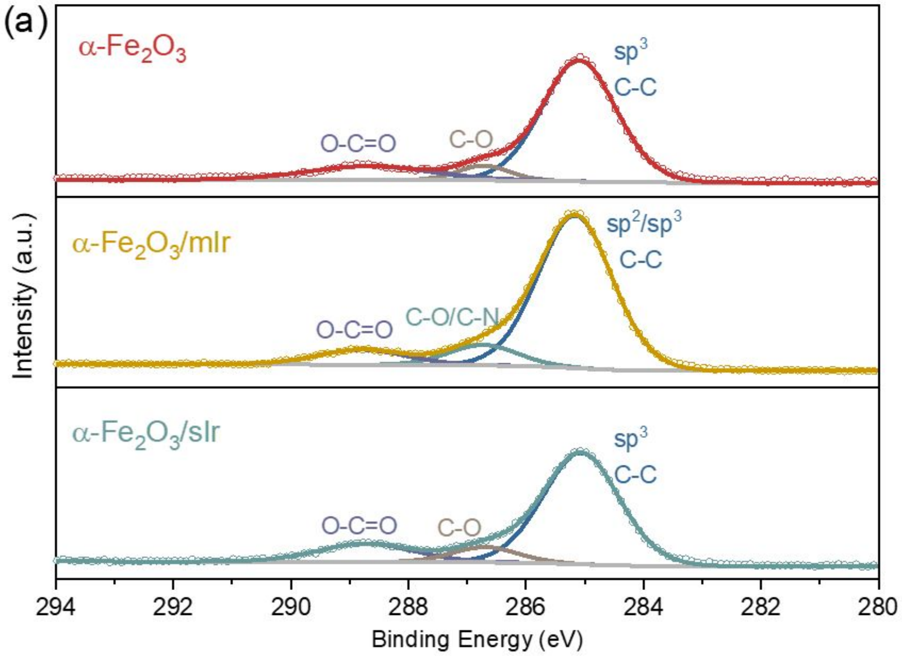

Figure S6. High-resolution XPS spectra of C 1s for α-Fe_2O_3, α-Fe_2O_3/mIr, and α-Fe_2O_3/sIr.

fs-ns and ms-s TAS absorption spectra of α-Fe_2O_3 and α-Fe_2O_3/sIr

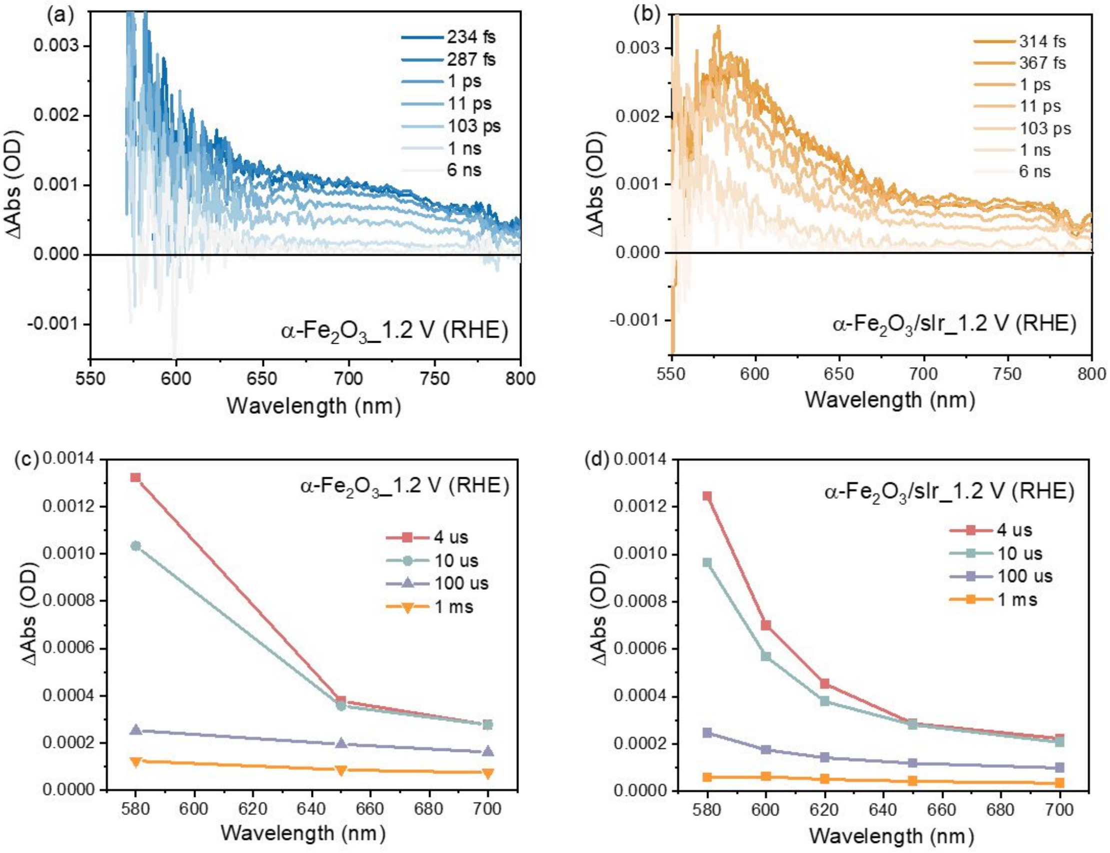

Figure S7. fs-ns transient absorption spectra of (a) α-Fe_2O_3 and (b) α-Fe_2O_3/sIr at 1.2 V vs. RHE; ms-s transient absorption spectra of (c) α-Fe_2O_3 and (d) α-Fe_2O_3 /sIr at 1.2 V *vs.* RHE.

fs-ns TAS kinetic profiles probed at 740 nm at different pump intensities

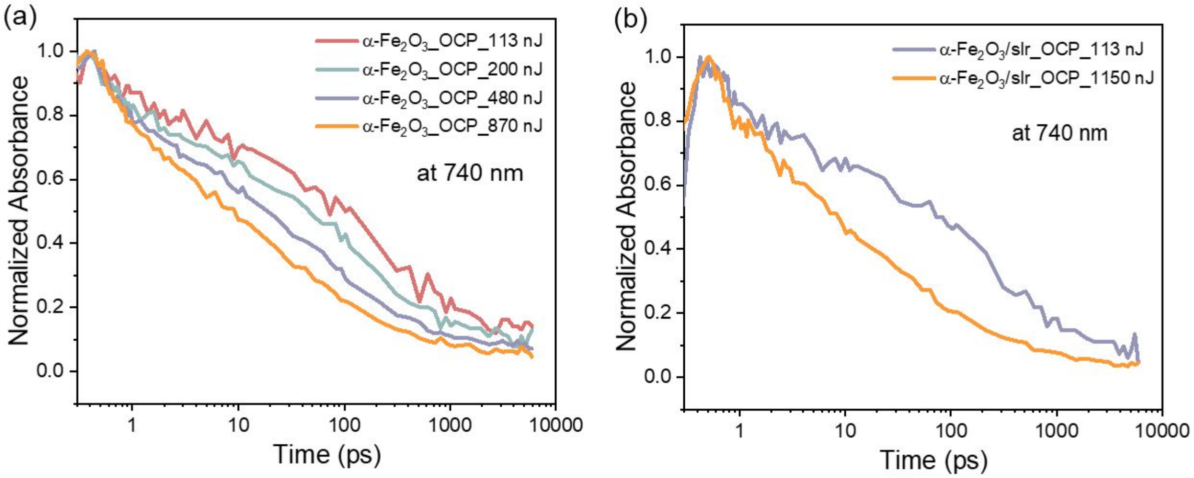

Figure S8. fs-ns transient kinetic spectra probed at 740 nm under various pump intensity for (a) α-Fe_2O_3 and (b) α-Fe_2O_3/sIr.

UV-Vis spectra and Mott-Schottky plots of α-Fe_2O_3 and α-Fe_2O_3/sIr

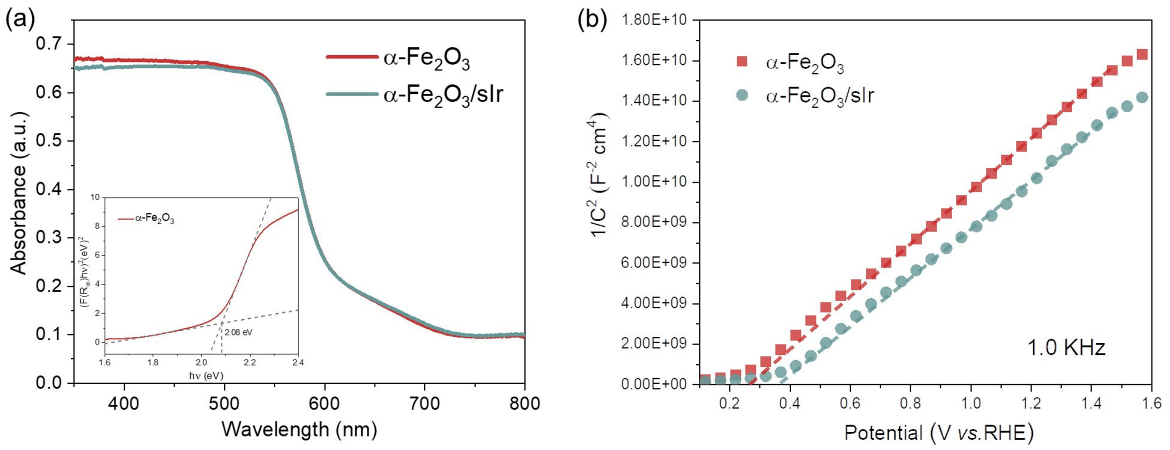

Figure S9. (a) UV-Vis spectra (inset: Tauc plots) and (b) Mott-Schottky results for α-Fe_2O_3 and α-Fe_2O_3/sIr.

DFT models of binding sites and binding energy for single Ir metal atoms on a α-Fe_2O_3 (110) surface

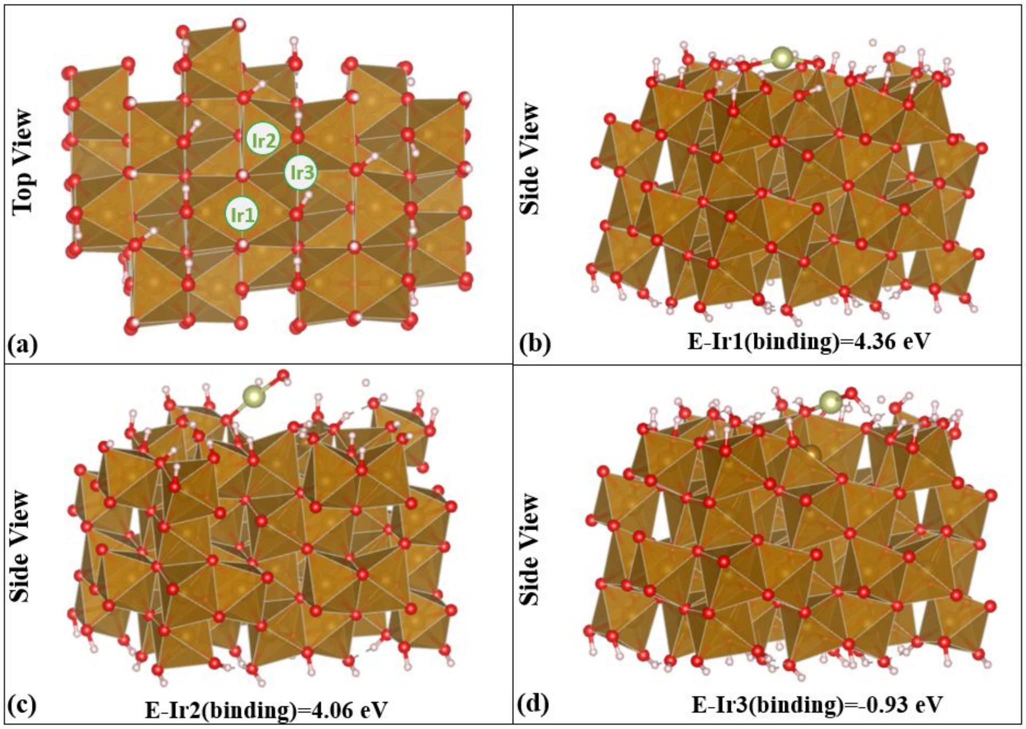

Figure S10. (a) Candidate binding sites for single Ir metal atoms on a α-Fe_2O_3 (110) surface; (b) The binding site and binding energy of Ir1 on α-Fe_2O_3 (110); (b) The binding site and binding energy of Ir2 on α-Fe_2O_3 (110)); (b)The binding site and binding energy of Ir3 on α-Fe_2O_3 (110).

Figure S10 shows the potential binding sites of sIr on α-Fe_2O_3 (110) facet There are in total three types of binding sites denoted as Ir1 (Figure S10a), Ir2 (Figure S10b), and Ir3 (Figure S10c), with binding energy was calculated to be of 4.36 eV, 4.06 eV and -0.93 eV, respectively, indicating Ir1 is the superior site for sIr solidly sitting on α-Fe_2O_3 (110). The Ir1 has the highest binding energy of 4.36 eV among them. When conducting DFT optimization, there is no structure deformation with Ir1 (Figure S10b) while Ir2 and Ir3 exhibit significant structure deformation as shown in Figure S10c and Figure S10d**.** Therefore, the structure of sIr on α-Fe_2O_3 (110) built by placing a Ir atom in bridge site (Ir1 site) between two terminal O atoms is used for DFT calculations in this work. The top view and side view of the created α-Fe_2O_3 (110) and α-Fe_2O_3/sIr (110) model can be seen in Figure S11.

DFT models for the structure of the hydroxyl terminated α-Fe_2O_3 (110) surface and α-Fe_2O_3/sIr (110) surface

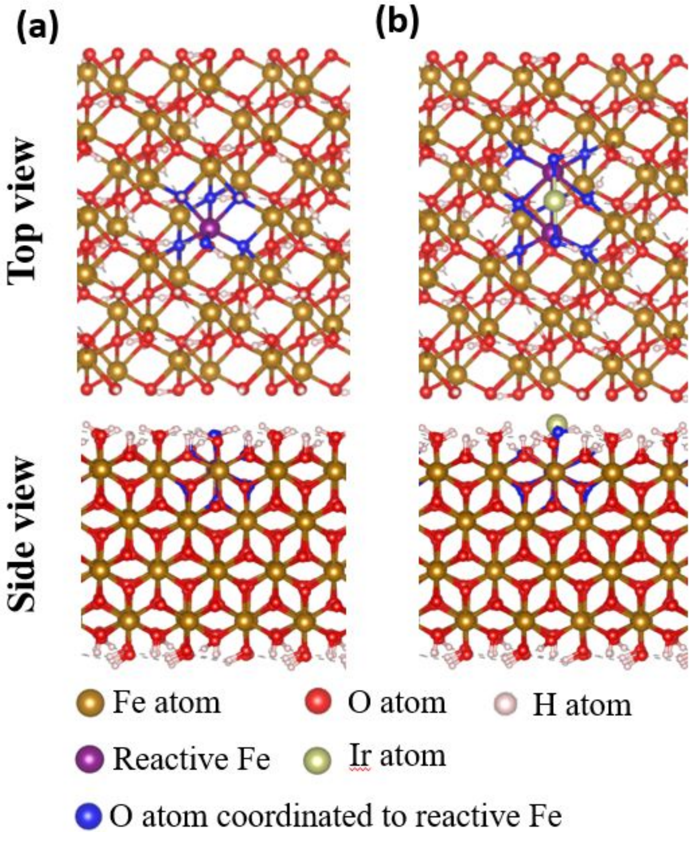

Figure S11. Structure of the hydroxyl terminated α-Fe_2O_3 (110) surface (a) and α-Fe_2O_3/sIr (110) surface (b).

Adsorption gibbs free energies of important intermediates on α-Fe_2O_3 (110) and α-Fe_2O_3/sIr (110)

Table S2 Adsorption gibbs free energies of important intermediates on α-Fe_2O_3 (110) and α-Fe_2O_3/sIr (110).

	Gad-OH* (eV)	Gad-OO* (eV)	Gad-OOH* (eV)	Gad-H_2O* (eV)
α-Fe_2O_3 (110)_Fe	-10.24	-8.90	-7.86	-7.97
α-Fe_2O_3/sIr (110)_Fe	-7.40	-5.07	-4.65	-5.41
α-Fe_2O_3/sIr (110)_Ir	-2.89	-0.81	-1.27	0.21

Bader charge analysis of metal centre and intermediates involved in the water oxidation reaction on α-Fe_2O_3/sIr (110)_Fe and α-Fe_2O_3/sIr (110)_Ir

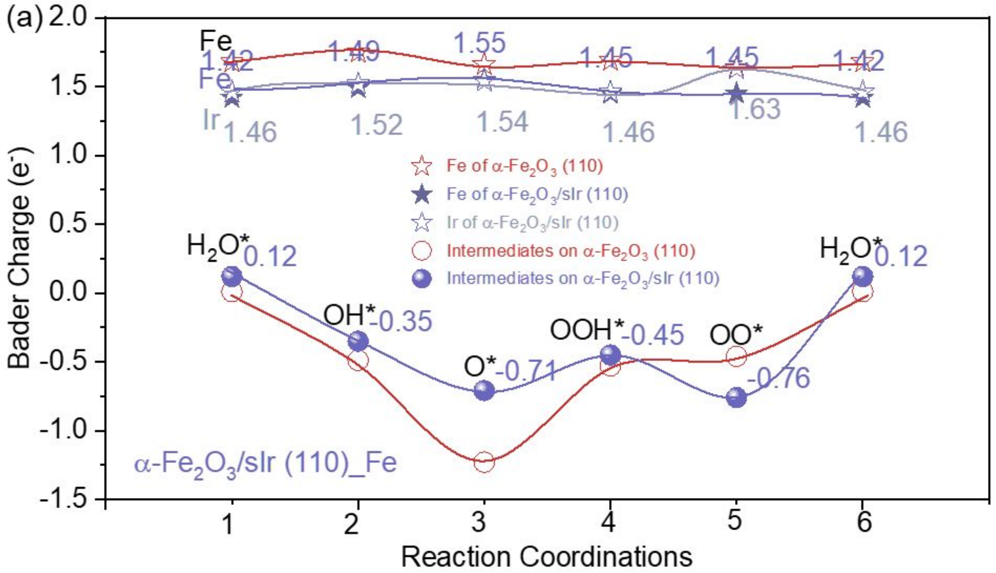

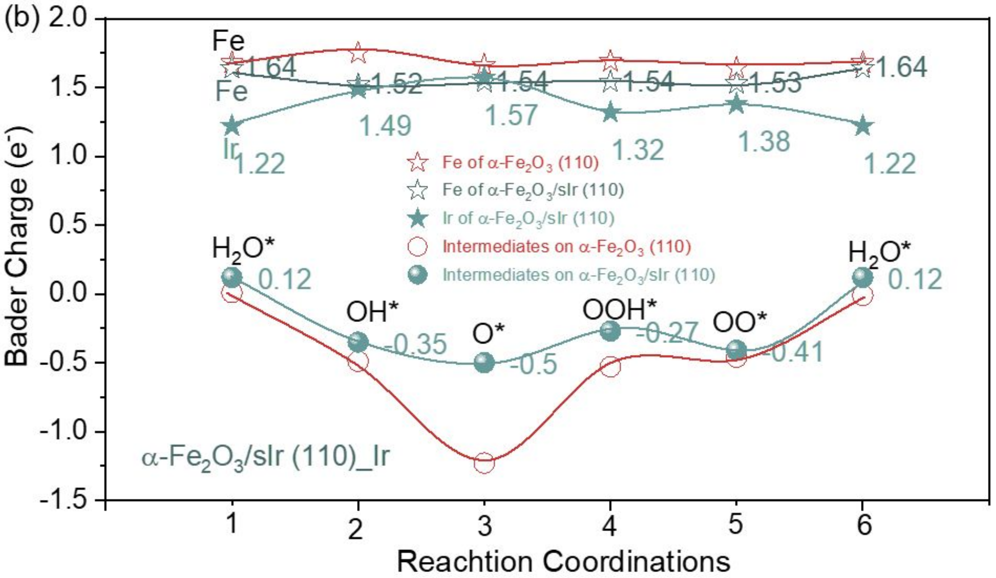

Figure S12. Bader charge analysis of metal centre and intermediates involved in the water oxidation reaction on (a) α-Fe_2O_3/sIr (110)_Fe and (b) α-Fe_2O_3/sIr (110)_Ir.

References

1 Jang, J.-W.; Du, C.; Ye, Y.; Lin, Y.; Yao, X.; Thorne, J.; Liu, E.; McMahon, G.; Zhu, J.; Javey, A.; Guo, J.; Wang, D. Enabling unassisted solar water splitting by iron oxide and silicon. *Nat. Commun.* **2015**, 1–5.

2 Zhao, Y.; Yang, K. R.; Wang, Z.; Yan, X.; Cao, S.; Ye, Y.; Dong, Q.; Zhang, X.; Thorne, J. E.; Jin, L.; Materna, K. L.; Trimpalis, A.; Bai, H.; Fakra, S. C.; Zhong, X.; Wang, P.; Pan, X.; Guo, J.; Flytzani-Stephanopoulos, M.; Brudvig, G. W.; Batista, V. S.; Wang, D. Stable iridium dinuclear heterogeneous catalysts supported on metal-oxide substrate for solar water oxidation. *Proc. Natl. Acad. Sci. U. S. A.* **2018,** *115*, 2902-2907.

3 Sheehan, S. W.; Thomsen, J. M.; Hintermair, U.; Crabtree, R. H.; Brudvig, G. W.; Schmuttenmaer, C. A. A molecular catalyst for water oxidation that binds to metal oxide surfaces. *Nat Commun* **2015,** *6,* 6469.

4 Tang, P.; Xie, H.; Ros, C.; Han, L.; Biset-Peiró, M.; He, Y.; Kramer, W.; Rodríguez, A. P.; Saucedo, E.; Galán-Mascarós, J. R.; Andreu, T.; Morante, J. R.; Arbiol, J. Enhanced photoelectrochemical water splitting of hematite multilayer nanowire photoanodes by tuning the surface state via bottom-up interfacial engineering. *Energy Environ. Sci.* **2017,** *10,* 2124-2136.

5 Kresse, G.; Furthmüller, J. Efficient iterative schemes for ab initio total-energy calculations using a plane-wave basis set. *Phys. Rev. B* **1996,** *54*, 11169.

6 Dudarev, S. L.; Botton, G. A.; Savrasov, S. Y.; Humphreys, C.; Sutton, A. P. Electron-energy-loss spectra and the structural stability of nickel oxide: An LSDA+ U study. *Phys. Rev. B* **1998,** *57,* 1505.

7 Kresse, G.; Joubert, D. From ultrasoft pseudopotentials to the projector augmented-wave method. *Phys. Rev. B* **1999,** *59***,** 1758.

8 Blöchl, P. E. Projector augmented-wave method. *Phys. Rev. B* **1994,** *50,* 17953-17979.

9 Perdew, J. P.; Burke, K.; Ernzerhof, M. Generalized Gradient Approximation Made Simple. *Phys. Rev. Lett.* **1996,** *77,* 3865-3868.

10 Wang, V.; Xu, N.; Liu, J. C.; Tang, G.; Geng, W.-T. VASPKIT: a pre-and post-processing program for VASP code. *arXiv preprint arXiv:1908.08269* (2019).

11 Nørskov, J. K.; Rossmeisl, J.; Logadottir, A.; Lindqvist, L.; Kitchin, J. R.; Bligaard, T.; Jonsson, H. Origin of the overpotential for oxygen reduction at a fuel-cell cathode. *J. Phys. Chem. B* **2004,** *108*, 17886-17892.

12 Nørskov, J. K.; Bligaard, T.; Logadottir, A.; Kitchin, J.; Chen, J. G.; Pandelov, S. Trends in the exchange current for hydrogen evolution. *J. Electrochem. Soc.* **2005,** *152*, J23.

13 Favaro, M.; Xiao, H.; Cheng, T.; Goddard, W. A.; Yano, J.; Crumlin, E. J. Subsurface oxide plays a critical role in CO2 activation by Cu (111) surfaces to form chemisorbed CO2, the first step in reduction of CO2. *Proc. Natl. Acad. Sci. U. S. A.* **2017,** *114*, 6706-6711.

14 Moss, B.; Wang, Q.; Butler, K. T.; Grau-Crespo, R.; Selim, S.; Regoutz, A.; Hisatomi, T.; Godin, R.; Payne, D. J.; Kafizas, A.; Domen, K.; Steier, L.; Durrant, J. R. Linking in situ charge accumulation to electronic structure in doped SrTiO3 reveals design principles for hydrogen-evolving photocatalysts. *Nat. Mater.* **2021**, *20*, 511-517.

15 Zhou, X.; Lan, J.; Liu, G.; Deng, K.; Yang, Y.; Nie, G.; Yu, J.; Zhi, L. Facet-mediated photodegradation of organic dye over hematite architectures by visible light. *Angew. Chem.-Int. Edit.* **2012**, 51, 178-182.

16 Patra, A. K.; Kundu, S. K.; Bhaumik, A.; Kim, D. Morphology evolution of single-crystalline hematite nanocrystals: magnetically recoverable nanocatalysts for enhanced facet-driven photoredox activity. *Nanoscale* **2016**, 8, 365-377.

17 Huang, X.; Hou, X.; Song, F.; Zhao, J.; Zhang, L. Facet-dependent Cr (VI) adsorption of hematite nanocrystals. *Environ. Sci. Technol.* **2016**, 50, 1964-1972.
